# The Development and Validation of Simplified Machine Learning Algorithms to Predict Prognosis of Hospitalized Patients With COVID-19: Multicenter, Retrospective Study

**DOI:** 10.2196/31549

**Published:** 2022-01-21

**Authors:** Fang He, John H Page, Kerry R Weinberg, Anirban Mishra

**Affiliations:** 1 Amgen Inc Center for Observational Research South San Francisco, CA United States; 2 Amgen Inc Digital Health & Innovation Thousand Oaks, CA United States; 3 Amgen Inc Center for Observational Research Thousand Oaks, CA United States

**Keywords:** COVID-19, predictive algorithm, prognostic model, machine learning

## Abstract

**Background:**

The current COVID-19 pandemic is unprecedented; under resource-constrained settings, predictive algorithms can help to stratify disease severity, alerting physicians of high-risk patients; however, there are only few risk scores derived from a substantially large electronic health record (EHR) data set, using simplified predictors as input.

**Objective:**

The objectives of this study were to develop and validate simplified machine learning algorithms that predict COVID-19 adverse outcomes; to evaluate the area under the receiver operating characteristic curve (AUC), sensitivity, specificity, and calibration of the algorithms; and to derive clinically meaningful thresholds.

**Methods:**

We performed machine learning model development and validation via a cohort study using multicenter, patient-level, longitudinal EHRs from the Optum COVID-19 database that provides anonymized, longitudinal EHR from across the United States. The models were developed based on clinical characteristics to predict 28-day in-hospital mortality, intensive care unit (ICU) admission, respiratory failure, and mechanical ventilator usages at inpatient setting. Data from patients who were admitted from February 1, 2020, to September 7, 2020, were randomly sampled into development, validation, and test data sets; data collected from September 7, 2020, to November 15, 2020, were reserved as the postdevelopment prospective test data set.

**Results:**

Of the 3.7 million patients in the analysis, 585,867 patients were diagnosed or tested positive for SARS-CoV-2, and 50,703 adult patients were hospitalized with COVID-19 between February 1 and November 15, 2020. Among the study cohort (n=50,703), there were 6204 deaths, 9564 ICU admissions, 6478 mechanically ventilated or EMCO patients, and 25,169 patients developed acute respiratory distress syndrome or respiratory failure within 28 days since hospital admission. The algorithms demonstrated high accuracy (AUC 0.89, 95% CI 0.89-0.89 on the test data set [n=10,752]), consistent prediction through the second wave of the pandemic from September to November (AUC 0.85, 95% CI 0.85-0.86) on the postdevelopment prospective test data set [n=14,863], great clinical relevance, and utility. Besides, a comprehensive set of 386 input covariates from baseline or at admission were included in the analysis; the end-to-end pipeline automates feature selection and model development. The parsimonious model with only 10 input predictors produced comparably accurate predictions; these 10 predictors (age, blood urea nitrogen, SpO_2_, systolic and diastolic blood pressures, respiration rate, pulse, temperature, albumin, and major cognitive disorder excluding stroke) are commonly measured and concordant with recognized risk factors for COVID-19.

**Conclusions:**

The systematic approach and rigorous validation demonstrate consistent model performance to predict even beyond the period of data collection, with satisfactory discriminatory power and great clinical utility. Overall, the study offers an accurate, validated, and reliable prediction model based on only 10 clinical features as a prognostic tool to stratifying patients with COVID-19 into intermediate-, high-, and very high-risk groups. This simple predictive tool is shared with a wider health care community, to enable service as an early warning system to alert physicians of possible high-risk patients, or as a resource triaging tool to optimize health care resources.

## Introduction

The COVID-19 pandemic has impacted more than 200 countries, claimed more than 3 million lives, presenting an urgent threat to global health. Under resource-constrained settings, a validated model using large-scale real-world data to predict COVID-19 prognosis can rapidly identify the individuals who are at risk of COVID-19 adverse outcomes and mortality, so they could benefit from early interventions.

Several studies have derived prognostic predictors for COVID-19; however, currently there are only few COVID-19 risk calculation tools with simplified predictors for stratification that leverage on a substantially large US electronic health record (EHR) data set of statistically meaningful size [1,2]. The Acute Physiology and Chronic Health Evaluation (APACHE) II score [3] has been widely used to predict in-hospital mortality, and has been found to predict mortality in patients with COVID-19, outperforming Sequential Organ Failure Assessment (SOFA) [4] and CURB-65 [5] scores in a retrospective study of 154 patients in China [6]. COVID-GRAM [2] is a web-based calculator to estimate the occurrence of ICU admission, mechanical ventilation, or death in hospitalized patients with COVID-19; it has been validated in a study of nearly 1600 patients in China. The Coronavirus Clinical Characterization Consortium (4C) Mortality Score [1] developed by the International Severe Acute Respiratory and Emerging Infections Consortium (ISARIC) World Health Organization (WHO) Clinical Characterisation Protocol UK (CCP-UK) study is a risk stratification tool to predict in-hospital mortality by categorizing patients at low, intermediate, high, or very high risk of death. Separately, an accurate, machine learning–based COVID-19 mortality prediction model has been developed based on data from the Mount Sinai Health System; however, its validation data set is limited in size [7].

The objective of this paper is to develop and validate simplified and parsimonious predictive algorithms, leveraging large size, near real-time real-world data as a risk stratification methodology to identify patients who are at heightened risk of (1) mortality; (2) ICU admission; (3) composite of invasive mechanical ventilation/extracorporeal membrane oxygenation (ECMO); (4) composite of acute respiratory distress syndrome (ARDS)/respiratory failure, which can be easily integrated into the hospital electronic medical record system as a risk stratification and triaging tool.

## Methods

### Data Source

This is a retrospective observational cohort analysis of multicenter, longitudinal, anonymized patient-level data from the Optum EHR COVID-19 database. It includes demographics, insurance status, medication prescription, vital signs, coded diagnoses, procedures, laboratory results, visits, encounters, and providers. Currently, there are 3,702,050 patients in the data release dated January 27, 2021. As deidentified data are used for the study, it was exempt from Institutional Review Board approval.

### Study Period

The study period was from February 1, 2020, to January 27, 2021. A baseline of up to 1 year prior to and including index date was used for assessment of demographics, lifestyle factors, and comorbidity at baseline. Patients were followed up to 28 days from admission, unless they were censored by in-hospital mortality or discharged.

### Participants

Study cohort consists of patients hospitalized with COVID-19 aged 18 and older, with a confirmed diagnosis or positive test of COVID-19 infection. A COVID-19 diagnosis was defined as the first occurrence on or after February 1, 2020, of any of the following: (1) positive result from SARS-CoV-2 viral RNA or antigen tests; (2) International Classification of Diseases, Tenth Revision, Clinical Modification (ICD-10-CM) diagnosis codes U07.1 (COVID-19, virus identified), J12.81 (pneumonia due to SARS-associated coronavirus), J12.89 (other viral pneumonia), or J80 (ARDS); and (3) ICD-10-CM code B97.29 (other coronavirus as the cause of disease) or B34.20 (coronavirus infection, unspecified) occurring on or before April 30, 2020. The expanded diagnosis code list, beyond COVID-19–specific diagnosis code (U07.1), was used because U07.1 was either unavailable (pre-April 2020) or was being implemented (April 2020), resulting in the use of alternative codes for COVID-19 in early pandemic. Other codes such as J20.3 (acute bronchitis due to coxsackievirus) were excluded due to very few uses (<10 patients) in the study period.

**Figure 1:**
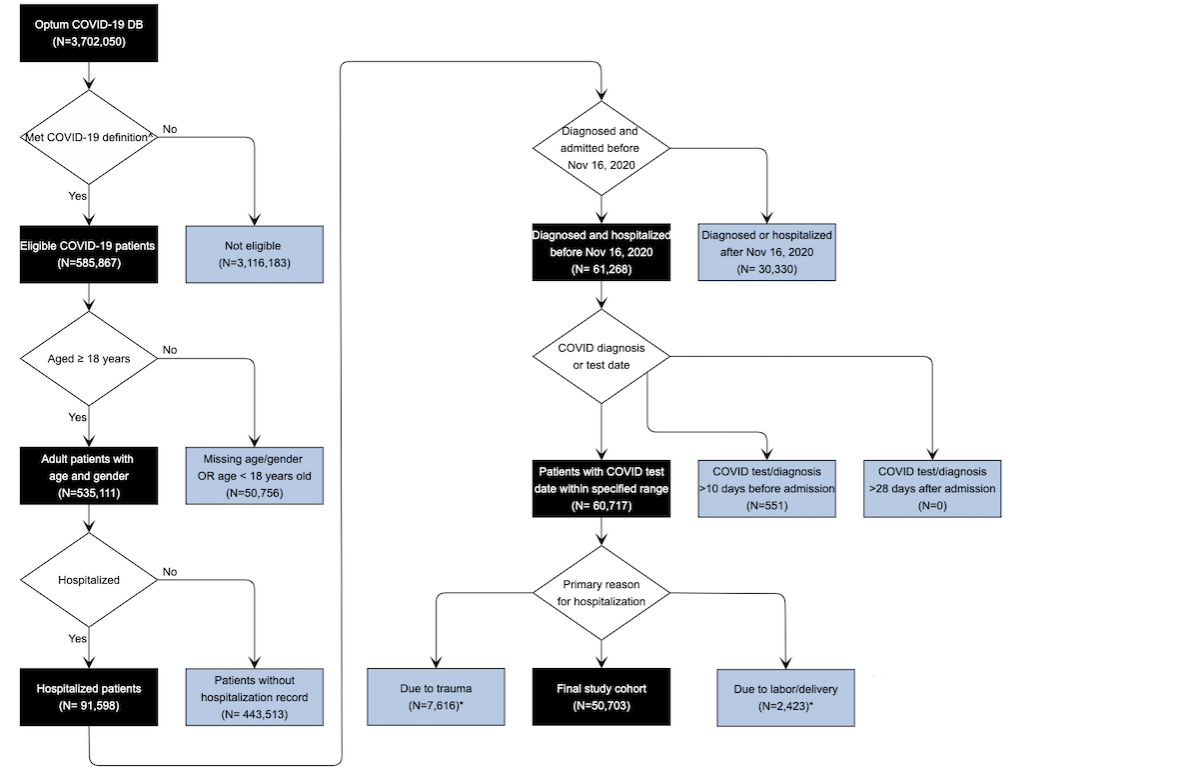
Patient attrition diagram. ^∧^With relevant COVID-19 diagnosis codes or tested positive for SARS-CoV-2. *Non-exclusive critera: overlapping was allowed.

Patients were excluded for any of the following: (1) missing age or sex; (2) under the age of 18; (3) diagnosis or procedure codes for labor and delivery during hospitalization; (4) diagnosis codes for trauma, injury, fracture, or poisoning during the first 2 days of hospitalization; (5) admitted to hospital more than 10 days prior to COVID-19 diagnosis or 28 days after COVID-19 diagnosis; (6) diagnosed or admitted to hospital after November 16, 2020, therefore with less than 10 weeks between their first COVID-19 diagnosis date or hospital admission date, and the last database refresh date (January 27, 2021; Figure 1). Additional sensitivity analysis was conducted between the final study cohort (n=50,703) and patients who tested positive for SARS-CoV-2 (n=38,277), a subset of the former.

### Sampling

In the final cohort that satisfied the study criteria (n=50,703), data from patients with an index date prior to September 7, 2020, were referred to as model development data set (n=35,840), which was randomly sampled without replacement using 28-day in-hospital mortality as stratification factor into 40% training data set (n=14,336), 30% validation data set (n=10,752) for hyperparameter tuning and threshold calculation, and 30% test data set (n=10,752). The sampling ratio is determined such that the validation or test data set alone can satisfy the sample size requirement; the minimum sample size is estimated to be 8605, assuming a predetermined sensitivity of 0.7 and the prevalence of all-cause mortality of 15% with 95% CI and maximum marginal error of estimate of 2.5% [8]. Furthermore, an independent validation consisting of patients with index date from September 7 to November 15, 2020, was referred to as postdevelopment prospective test data set (n=14,863).

### Index Date

The index date was defined as hospital admission date.

### Sample Size

The initial anonymized data for 3,702,050 patients from 885,677 providers and 2465 delivery networks for the study period February 1, 2020, to January 27, 2021, were transferred from Optum, among which 585,867 patients were diagnosed or tested positive for SARS-CoV-2 infection.

### Outcome

The outcomes were 28-day in-hospital (1) all-cause mortality; (2) ICU admission; (3) composite of invasive mechanical ventilation or ECMO; (4) composite of ARDS and respiratory failure. These were assessed as dichotomous outcomes and individually modeled. Outcome-specific exclusions were applied as appropriate to include only incident outcomes.

### Covariates

A total of 386 study covariates (with a minimum 70% [35,493/50,703 patients] coverage among study cohort) consisting of patients’ baseline demographics (age, sex, census division, insurance status, race, ethnicity), lifestyle factors (smoking status, BMI), comorbidities (including atrial fibrillation cancer history, cerebrovascular disease, chronic kidney disease stage I-V, chronic obstructive pulmonary disease, coronary artery disease, Type I/II diabetes mellitus, HIV, stroke, etc.), baseline medication (including antidiabetics, anticoagulants, antihypertensives, antiplatelets, steroids, etc.) within 12 months prior to index date, vital signs (blood pressures, heart rate, pulse, respiration rate, temperature), laboratory values (including albumin, alanine transaminase, aspartate aminotransferase, total bilirubin, B-type natriuretic peptide, blood urea nitrogen (BUN), chloride, creatinine, C-reactive protein, D-dimer, fibrinogen, hemoglobin, lymphocyte, monocyte, neutrophil, oxygen saturation platelet count, arterial blood pH, etc.), and treatment (including diuretics, disease-modifying antirheumatic drugs, steroids, etc.) administered on the day of hospital admission were included in the analysis. Concretely, baseline medication, comorbidity, and postadmission treatment were expressed as dichotomous variables; categorical variables were converted to dummy variables; numerical variables were used without standardization, unless when fitting to penalized (Lasso or Ridge) logistic regression models, while numerical covariates were normalized using a min–max standardization to speed up convergence.

### Missing Data

One of the challenges of working with real-world data is the missing covariates. Assuming covariates are missing at random, multiple imputation by chained equations via random forest [9] was used to impute covariates with missing values. Ten complete data sets each with 10 iterations were imputed with predictive mean matching using available covariates while excluding the outcome variables. The prediction performances of sparsity-aware models (XGB [10]) between imputed and nonimputed data set were compared in the sensitivity analysis.

Given the intention to develop an algorithm of great relevance to as many patients as possible, we have restricted the model input to covariates with a minimum of 70% coverage in the study cohort. Overall, the proportion of missing values among the vital and laboratory variables ranges from 10.44% (5295/50,703) to 99.36% (50,381/50,703) out of 50,703 patients; 45 of 431 variables were not included as input to the model due to more than 30% (15,211/50,703) of missingness (Multimedia Appendices 1 and 2). Sensitivity analysis was conducted to evaluate whether inclusion of additional covariates with higher degrees of missingness (ie, varying the cutoffs from 10% to 90%) aids in improving model performance, though it may increase the sensitivity of the models to biases due to nonignorable missingness in the data.

### Model Development

We have applied a systematic approach to model development and validation. A framework of 6 machine learning algorithms (XGB [10,11], penalized logistic regression [12,13] with Lasso [14] or Ridge loss [11], random forest [11,15,16], decision tree [17], and LightGBM [18]) has been adopted to develop interpretable models to predict the prognosis of COVID-19.

In the preliminary analysis, the most performant algorithm was selected from the candidate algorithms; prior to model training, hyperparameter optimization via grid search, ranging from 96 to 243 folds, was performed on 6 candidate algorithms individually for full and simplified models. The full model uses all the available 386 input features after extraction and transformation in preliminary analysis, while the simplified model recursively eliminates the aforementioned input to yield a maximum of 20 variables [19]. The algorithm with best performance (area under the receiver operating characteristic curve [AUC], Brier score [20], and calibration [21]) on the test data set was selected for the final analysis.

In the final analysis, model input is further iteratively reduced to a maximum of 5 variables with a step size of 1; 100 individual runs were performed at each step, with retuned model parameters every 5 steps. The selected features were pooled and plotted in frequency heatmap with the corresponding AUC.

The model performance is evaluated against outcome variables in the test and postdevelopment prospective test data sets via AUC, Brier score, and calibration curve. The 95% CIs for AUC and Brier score were calculated based on percentiles from bootstrapped resampling with replacement (bootstrap sample size = 2000) without bias correction or acceleration [22]. The calibration curves (number of discretized bins = 10) were plotted for all the runs.

### Model Validation

Rigorous validation analysis was performed to ensure robustness and reliability of the predictions. Both full and simplified models of 6 candidate algorithms were trained and validated during the model development phase with data from February 1 to September 6, where the test data set was held out from model training and used solely for reporting the performance. Furthermore, the model has been additionally validated externally, using the postdevelopment prospective test data set collected from September 7 to November 15, 2020, to demonstrate consistent model performance through the subsequent wave of the pandemic. Model discrimination was performed on the imputed test data set by assessing AUC on the stratified analysis by sex, age, and racial groups.

### Model Benchmark

The performance of the risk prediction models has been benchmarked to (1) the baseline model and (2) published COVID-19 prognostic scores. The baseline model was developed using XGB with optimized hyperparameters on age and sex only. Evaluation metrics including AUC, sensitivity, specificity, and decision curve analysis were assessed to compare the performance and utility of prognostic scores (APACHE II [3,23], CURB-65 [5,24,25], E-CURB [26], The National Early Warning [NEWS] 2 score [25,27-29], Respiratory Rate-Oxygenation [ROX] index [29,30], ISARIC 4C mortality score [1,25]). AUC is reported based on complete case data from test and postdevelopment prospective test data sets, and no imputation was performed.

### Predictors

Feature importance is ranked by Shapley values [31] from test and postdevelopment prospective test data sets in the SHapley Additive exPlanations (SHAP) summary plot. Shapley value calculates fair contribution and the extent of predictors toward the model output [32]. It measures feature importance by the magnitude and the direction of contributions. The dependence between model prediction and age is plotted with age on the x-axis and its impact on prediction represented by Shapley value on the y-axis for every patient, colored by the magnitude of a second feature (BUN, respiration rate, pulse, lymphocyte count) individually.

### Receiver Operating Characteristic Curve Analysis

We adopted two approaches in determining the optimal threshold on the receiver operating characteristic curve. Assuming the sensitivity and specificity were weighted equally without ethical, cost, and prevalence constraints, the optimal cutoff is at the location where the Youden index (sum of specificity and sensitivity – 1) is maximized at the test data set [33-36]. This approach relies solely on the predictive accuracy of a model, and consequences of the predictions (ie, cost of false positives and false negatives) are not considered. In the second approach, clinical utility–based decision theory was used in developing a cost-sensitive threshold, where it builds in disease prevalence and costs of false positive and false negatives of specific diagnostic scenario [33,37].

Decision curve analysis assists in clinical judgment and comparison about the relative value of benefits associated with the use of a clinical prediction tool [38,39]. The standardized net benefit of full model, simplified model (with 10 input variables), and selected benchmark prognostic scores was calculated and plotted across probabilities. The benchmark models that use point scores were calibrated to test data prior to decision curve analysis.

## Results

### Patient Characteristics

**Figure 2:**
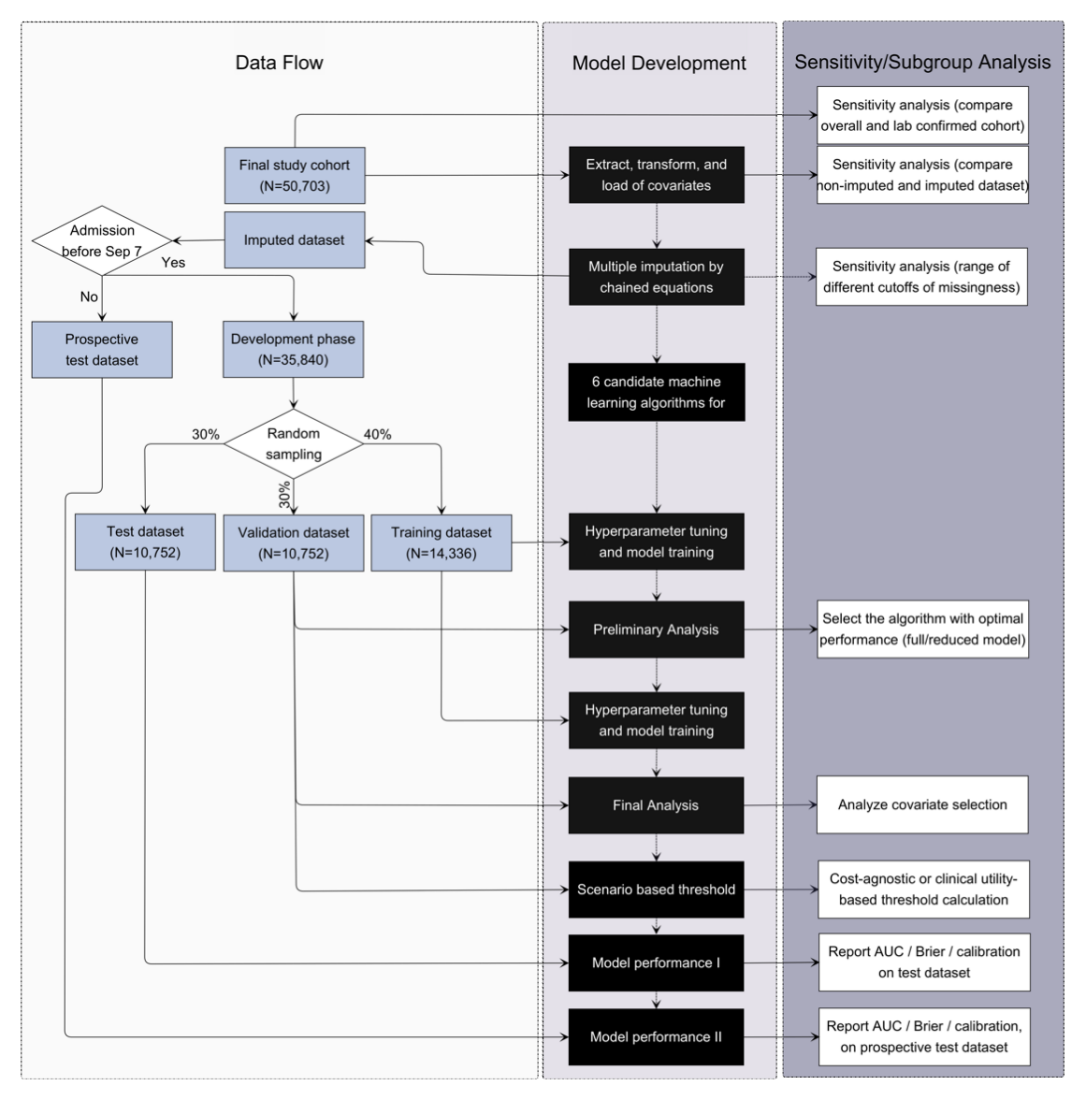
Model development and validation framework including data sampling and corresponding sensitivity analyses.

Figure 1 shows patient attrition flowchart, and the workflow of model development and validation is in Figure 2. Patients’ baseline and clinical characteristics at admission are summarized in Table 1. Validation and test data sets are largely homogeneous to the training data set; however, the postdevelopment prospective test data set that was collected later in the pandemic from September to November presents more differences in geographic locations (a decline in the proportion of patients in Middle Atlantic from 22.69% [8132/35,840] to 8.68% [1290/14,863] after September 7, and an increase in West North Central from 9.83% [3523/35,840] to 24.18% [3594/14,863]) and racial distribution (the proportion of White increased from 53.87% [19,308/35,840] to 72.88% [10,832/14,863]). However, the overall mortality remains consistent. Hypertension (57.55% [29,179/50,703]), obesity (47.51% [24,089/50,703]), diabetes mellitus (34.44% [17,461/50,703]), chronic kidney disease (19.79% [10,033/50,703]), and coronary artery disease (17.74% [8996/50,703]) were the common comorbidities among the cohort.

The study cohort is defined as hospitalized adult patients with COVID-19 who were either diagnosed with relevant diagnosis codes or tested positive for SARS-CoV-2 viral RNA or antigen tests. In the subgroup analysis of patients who tested positive for SARS-CoV-2, model performances of these 2 groups (ie, overall cohort and tested positive subgroup) were largely similar with less than 1% difference in AUC across all outcomes for full and simplified models (Multimedia Appendix 3).

**Table 1:** Demographic and clinical characteristics of hospitalized patients with COVID-19 at baseline and admission.

Characteristic		Training data set (n=14,336)	Validation data set (n=10,752)	Test data set (n=10,752)	Prospective test data set (n=14,863)	
Mean (SD) age at baseline, years		60.9 (17.2)	60.9 (17.2)	60.8 (17.1)	63.8 (16.8)	
**Distribution, n (%)**						
	18-34	1231 (8.59)	920 (8.56)	911 (8.47)	1015 (6.83)	
	35-49	2383 (16.62)	1780 (16.56)	1840 (17.11)	1893 (12.74)	
	50-64	4337 (30.25)	3193 (29.70)	3293 (30.63)	4110 (27.65)	
	65-74	2922 (20.38)	2296 (21.35)	2141 (19.91)	3325 (22.37)	
	75-84	2165 (15.10)	1589 (14.78)	1606 (14.94)	2943 (19.80)	
	85+	1298 (9.05)	974 (9.06)	961 (8.94)	1577 (10.61)	
**Sex at baseline, n (%)**						
	Male	7473 (52.13)	5619 (52.26)	5629 (52.35)	7645 (51.44)	
	Female	6863 (47.87)	5133 (47.74)	5123 (47.65)	7218 (48.56)	
**Race at baseline, n (%)**						
	African American	3466 (24.18)	2669 (24.82)	2668 (24.81)	1867 (12.56)	
	Asian	368 (2.57)	268 (2.49)	276 (2.57)	216 (1.45)	
	White	7779 (54.26)	5734 (53.33)	5795 (53.90)	10,832 (72.88)	
	Other/Unknown	2723 (18.99)	2081 (19.35)	2013 (18.72)	1948 (13.11)	
**Census division at baseline, n (%)**						
	East North Central	3778 (26.35)	2942 (27.36)	2908 (27.05)	4174 (28.08)	
	East South Central	1010 (7.05)	708 (6.58)	754 (7.01)	1205 (8.11)	
	Middle Atlantic	3221 (22.47)	2488 (23.14)	2423 (22.54)	1290 (8.68)	
	Mountain	496 (3.46)	355 (3.30)	363 (3.38)	923 (6.21)	
	New England	1042 (7.27)	705 (6.56)	763 (7.10)	769 (5.17)	
	Pacific	475 (3.31)	331 (3.08)	317 (2.95)	345 (2.32)	
	South Atlantic/West South Central	2454 (17.12)	1802 (16.76)	1810 (16.83)	2120 (14.26)	
	West North Central	1396 (9.74)	1067 (9.92)	1060 (9.86)	3594 (24.18)	
	Other/Unknown	464 (3.24)	354 (3.29)	354 (3.29)	443 (2.98)	
BMI at baseline (kg/m^2^), mean (SD)		31.0 (8.5)	30.9 (8.3)	31.2 (8.6)	31.6 (8.7)	
**Distribution, n (%)**						
	Underweight	352 (2.46)	235 (2.19)	221 (2.06)	304 (2.05)	
	Healthy weight	2526 (17.62)	1873 (17.42)	1833 (17.05)	2283 (15.36)	
	Overweight	3697 (25.79)	2878 (26.77)	2838 (26.40)	3679 (24.75)	
	Obese	3041 (21.21)	2247 (20.90)	2344 (21.80)	3228 (21.72)	
	Morbidly obese	3679 (25.66)	2739 (25.47)	2742 (25.50)	4069 (27.38)	
	Unknown	1041 (7.26)	780 (7.25)	774 (7.20)	1300 (8.75)	
**Comorbidity at baseline^a^, n (%)**						
	Cerebrovascular disease	676 (4.72)	502 (4.67)	501 (4.66)	894 (6.01)	
	Chronic kidney disease	2808 (19.59)	2058 (19.14)	2040 (18.97)	3127 (21.04)	
	Congestive heart failure	2137 (14.91)	1534 (14.27)	1553 (14.44)	2369 (15.94)	
	Coronary artery disease	2430 (16.95)	1797 (16.71)	1800 (16.74)	2969 (19.98)	
	Diabetes mellitus	4831 (33.70)	3636 (33.82)	3586 (33.35)	5408 (36.39)	
	Hypertension	8173 (57.01)	6091 (56.65)	6063 (56.39)	8852 (59.56)	
	Solid tumor	830 (5.79)	606 (5.64)	619 (5.76)	1052 (7.08)	
	Transplant history	28 (0.20)	16 (0.15)	20 (0.19)	12 (0.08)	
**28-day outcomes, n (%)**						
	All-cause mortality	1769 (12.34)	1326 (12.33)	1327 (12.34)	1782 (11.99)	
	Intensive care unit admission	2813 (19.62)	2181 (20.28)	2148 (19.98)	2422 (16.30)	
	Acute respiratory distress syndrome (respiratory failure)	7276 (50.75)	5500 (51.15)	5384 (50.07)	7009 (47.16)	
	Extracorporeal membrane oxygenation (mechanical ventilation)	1962 (13.69)	1535 (14.28)	1498 (13.93)	1483 (9.98)	
**Vitals at admission, median (10th-90th percentile)**						
	Diastolic blood pressure (mmHg)^b^	73.0 (56.0-90.0)	73.0 (56.0-90.0)	73.0 (56.0-90.0)	73.0 (56.0-90.0)	
	Systolic blood pressure (mmHg)^b^	125.0 (100.0-154.0)	125.0 (101.0-155.0)	125.0 (101.0-154.0)	128.0 (103.0-159.0)	
	Pulse (bpm)^b^	85.0 (64.0-110.0)	85.0 (64.0-110.0)	85.0 (64.0-110.0)	81.0 (61.0-107.6)	
	Respiratory rate (breaths/minute)^b^	19.0 (16.0-28.0)	19.0 (16.0-28.0)	19.0 (16.0-28.0)	18.0 (16.0-25.0)	
	Temperature (^o^C)^b^	36.8 (36.3-37.9)	36.8 (36.3-37.9)	36.8 (36.3-37.8)	36.7 (36.2-37.7)	
**Laboratory values^a^ at admission, median (10th percentile-90th percentile)**						
	Alkaline phosphatase (IU/L)	77.0 (49.0-137.0)	76.0 (49.0-136.0)	76.0 (48.0-135.0)	78.0 (50.0-134.0)	
	Alanine aminotransferase (IU/L)	28.0 (12.0-79.0)	29.0 (12.0-80.0)	28.0 (12.0-79.0)	27.0 (12.0-68.0)	
	Aspartate aminotransferase (IU/L)	37.0 (18.0-95.0)	36.0 (18.0-97.0)	36.0 (18.0-95.0)	34.0 (18.0-80.0)	
	Albumin (g/dL)	3.5 (2.7-4.2)	3.6 (2.7-4.2)	3.6 (2.7-4.2)	3.6 (2.8-4.2)	
	Anion gap (mEq/L)	12.0 (7.0-17.0)	12.0 (7.0-17.0)	12.0 (7.0-17.0)	12.0 (7.0-16.0)	
	Blood urea nitrogen (mg/dL)	16.0 (8.0-47.0)	17.0 (8.0-46.0)	16.0 (8.0-47.0)	18.0 (9.0-44.0)	
	Bicarbonate (mmol/L)	24.0 (19.0-29.0)	24.0 (19.0-29.0)	24.0 (19.0-29.0)	24.0 (19.0-29.0)	
	Bilirubin total (mg/dL)	0.6 (0.3-1.2)	0.6 (0.3-1.2)	0.6 (0.3-1.2)	0.6 (0.3-1.1)	
	C-reactive protein (mg/dL)	85.0 (10.3-229.0)	82.2 (11.0-218.0)	82.0 (10.2-220.0)	73.0 (10.0-206.6)	
	Chloride (mmol/L)	101.0 (94.0-108.0)	101.0 (94.0-108.0)	101.0 (94.0-108.0)	101.0 (94.0-107.0)	
	Glucose (mg/dL)	120.0 (91.0-242.0)	121.0 (92.0-236.0)	121.0 (92.0-240.6)	122.0 (91.2-244.0)	
	Hemoglobin (g/dL)	13.2 (10.0-15.5)	13.2 (10.1-15.6)	13.2 (10.2-15.7)	13.2 (10.1-15.6)	
	Lymphocyte (%)	14.1 (5.4-30.0)	14.8 (5.6-30.7)	14.6 (5.8-30.2)	14.1 (5.3-30.0)	
	Monocyte (%)	7.1 (3.1-12.9)	7.0 (3.2-12.6)	7.1 (3.2-12.7)	7.8 (3.6-13.1)	
	Neutrophil (%)	75.8 (57.0-88.0)	75.0 (57.0-88.0)	75.2 (57.0-88.0)	75.0 (57.0-88.0)	
	Platelet count (x10^9^/L)	210.0 (125.0-351.0)	210.0 (127.0-348.0)	211.0 (126.0-351.0)	205.0 (124.0-335.0)	
	Potassium (mmol/L)	3.9 (3.3-4.8)	3.9 (3.3-4.8)	3.9 (3.3-4.8)	3.9 (3.3-4.7)	
	Protein total (g/dL)	7.2 (6.2-8.2)	7.2 (6.2-8.2)	7.3 (6.2-8.2)	7.1 (6.2-8.0)	
	Red cell distribution width coefficient of variation (%)	13.9 (12.4-17.0)	13.8 (12.4-16.9)	13.8 (12.4-17.0)	13.8 (12.4-16.7)	
	Sodium (mmol/L)	136.0 (130.0-141.0)	136.0 (131.0-142.0)	136.0 (131.0-141.0)	136.0 (131.0-141.0)	
	Oxygen saturation pulse oximeter (%)	96.0 (91.0-99.0)	96.0 (90.0-99.0)	96.0 (91.0-99.0)	95.0 (90.0-99.0)	
	Oxygen saturation pulse oximeter^b^ (%)	95.0 (87.0-99.0)	95.0 (87.0-99.0)	95.0 (87.0-99.0)	95.0 (87.0-99.0)	
	Oxygen saturation pulse oximeter^c^ (%)	93.0 (84.0-97.0)	93.0 (84.0-97.0)	93.0 (84.0-97.0)	92.0 (83.0-97.0)	
	White blood cell count (x10^9^/L)	7.1 (4.0-14.1)	7.1 (4.0-13.9)	7.0 (4.0-13.8)	6.9 (3.9-13.5)	

^a^Non-exhaustive list.

^b^First measurement on the day of hospital admission.

^c^Minimum measurement on the day of hospital admission.

### Model Performance

**Figure 3:**
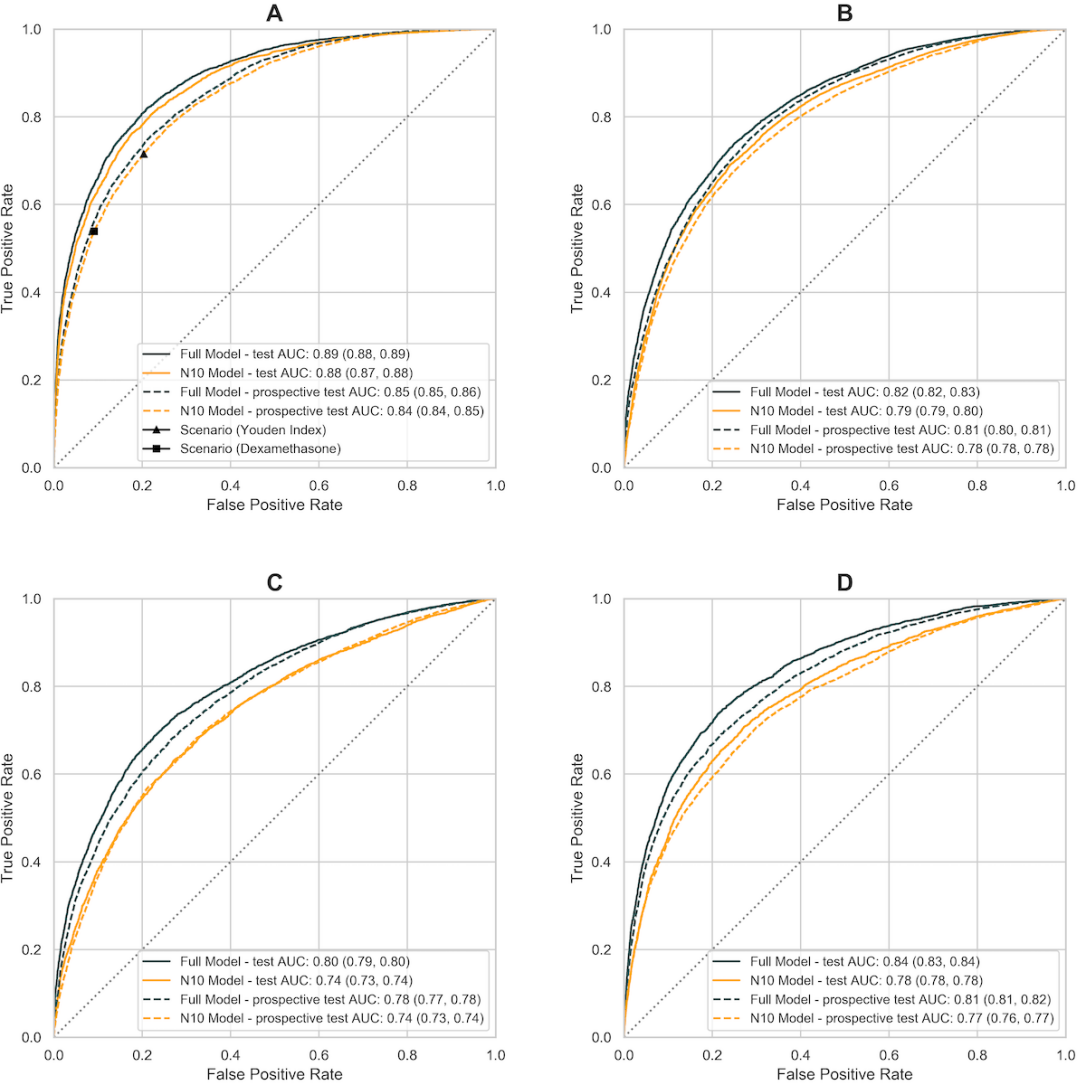
Receiver operating characteristics (AUROC) curves on four prediction outcomes in final analysis: (a) all-cause mortality; (b) respiratory failure including ARDS; (c) ICU admission; (d) invasive mechanical ventilation including ECMO. Full model is colored in black, parsimonious model with ten input variables is colored in orange. Solid line represents model performance on test dataset (n=10,752); dashed line represents post-development prospective test dataset (n=14,863). ARDS: acute respiratory distress syndrome. ECMO: extracorporeal membrane oxygenation.

**Figure 4:**
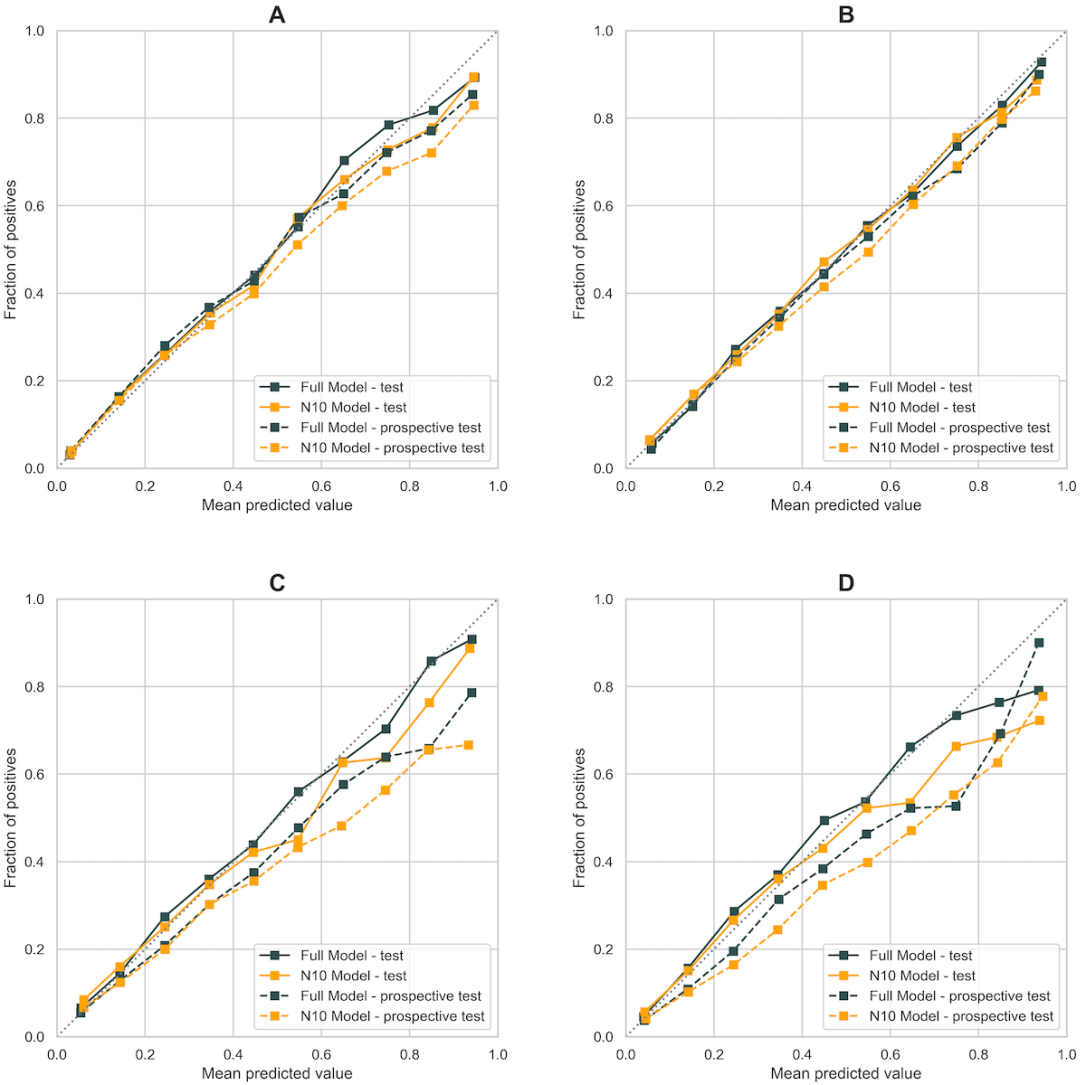
Calibration curve (number of bins = 10) on four prediction outcomes in final analysis: (a) all-cause mortality; (b) respiratory failure including ARDS; (c) ICU admission; (d) invasive mechanical ventilation including ECMO. Full model is colored in black, parsimonious model with ten input variables is colored in orange. Solid line represents calibration on test dataset (n=10,752); dashed line represents calibration on post-development prospective test dataset (n=14,863). ARDS: acute respiratory distress syndrome. ECMO: extracorporeal membrane oxygenation.

We have adopted a systematic framework of model development, including a variety of tree-, boosting-, and ensemble-based machine learning models, combined with rigorous validation on statistically meaningful sample size. The model performances (AUC and Brier score) on test and prospective test data sets are summarized in Table 2 and Figure 3. AUC is a widely used metric for performance measurement of classification; Brier score is a proper scoring rule, measuring mean squared error between prediction and outcome, impacted by both discrimination and calibration. Calibration of the algorithm is further assessed by plotting the predicted proportion against the observed proportion of outcome in each decile of risk (Figure 4).

**Table 2:** Summary of model performances (AUC^a^ and Brier Score) on test data set and postdevelopment prospective test data set in the final analysis. The full model uses all the available 210 covariates with less than 30% (15,211/50,703) missingness (excluding postadmission treatment) among the study cohort (n=50,703); the parsimonious N10 model only uses 10 predictors prefiltered from the automatic predictor selection.

Outcome and model		AUC^a^ (95% CI)		Brier score (95% CI)		
		Test data set, %	Prospective test data set, %	Test data set	Prospective test data set	
**All-cause mortality**						
	Full model	88.7 (88.4-89.0)	85.4 (85.1-85.7)	0.071 (0.070-0.072)	0.079 (0.078-0.080)	
	N10 model	87.6 (87.2-87.9)	84.3 (84.0-84.6)	0.074 (0.073-0.075)	0.081 (0.080-0.081)	
**Intensive care unit admission**						
	Full model	79.7 (79.4-80.1)	77.7 (77.3-78.0)	0.123 (0.122-0.124)	0.115 (0.114-0.115)	
	N10 model	73.6 (73.2-74.0)	73.5 (73.2-73.9)	0.138 (0.137-0.139)	0.123 (0.122-0.124)	
**Respiratory failure^b^**						
	Full model	82.3 (82.0-82.5)	80.7 (80.5-80.9)	0.172 (0.171-0.173)	0.180 (0.179-0.181)	
	N10 model	79.5 (79.2-79.7)	78.1 (77.9-78.3)	0.185 (0.184-0.186)	0.192 (0.191-0.193)	
**Mechanical ventilation^c^**						
	Full model	83.6 (83.3-84.0)	81.1 (80.8-81.5)	0.090 (0.089-0.091)	0.074 (0.074-0.075)	
	N10 model	78.1 (77.7-78.5)	76.6 (76.2-77.1)	0.101 (0.100-0.101)	0.081 (0.081-0.082)	

^a^AUC: area under the receiver operating characteristic curve.

^b^Refers to composite of respiratory failure and acute respiratory distress syndrome.

^c^Refers to composite of invasive mechanical ventilation and extracorporeal membrane oxygenation.

The model predicts 28-day in-hospital mortality accurately (AUC 0.88, 95% CI 0.87-0.88 on the test data set) and reliably through the second wave of pandemic (AUC 0.84, 95% CI 0.84-0.85 on the prospective test data set). Given this data set was acquired later in time from September to November and more likely to suffer from data lag, the completeness and accuracy of outcome data are hypothesized to contribute to the decrease in model performance; a subgroup analysis on patients with the complete clinical features shows an improved performance (AUC 0.89, 95% CI 0.88-0.90; Table 3).

We also examined discriminatory capacity in subgroups stratified by sex, race, and age group separately. It predicts all-cause mortality similarly among men (AUC 0.84, 95% CI 0.84-0.84) and women (AUC 0.84, 95% CI 0.84-0.85) and is marginally more predictive among Asians (AUC 0.86, 95% CI 0.85-0.87) compared with African Americans (AUC 0.83, 95% CI 0.83-0.84) and Whites (AUC 0.84, 95% CI 0.84-0.84). Given age is an important predictor, the model is more sensitive toward elderly cohort (more accurately ruling out negative cases) and conversely more specific toward younger cohort (more accurately ruling in the positive cases).

### Algorithm Selection

In the preliminary analysis, all the candidate algorithms perform comparably on test and prospective test data sets, with less than 3% difference in AUC for all outcomes between full and simplified models (Multimedia Appendix 4). Of the 6 candidate machine learning algorithms, boosting-based algorithms (XGB [10] and LightGBM [18]) performed consistently better [40] for both full and preliminary simplified models (n=20) with less computation time and produced well-calibrated probabilities (Multimedia Appendices 5 and 6); XGB was selected given it has been validated in a similar approach [7,41,42]. With adequate model calibration and low Brier score, no adjustment or calibration was subsequently performed.

### Predictor Selection

Predictors were selected in the development pipeline; specifically, 100 individual runs of recursive predictor elimination are pooled at each step between 5- and 20-input model with an increment of 1. The selection of predictors was analyzed in the frequency heatmap (Multimedia Appendix 7) and automated from the pipeline while nonmodifiable factors such as diagnosis month or census division were precluded.

With only 10 predictors, the final parsimonious model (N10) still predicts COVID-19 adverse outcomes accurately and similarly to the full model (Table 2); for instance, the final parsimonious model consisting of age, systolic and diastolic blood pressures, respiration rate, pulse, temperature, BUN, SpO_2_, albumin, and presence of any major cognitive disorder (including dementia, Parkinson disease, and Alzheimer disease) as input predicts all-cause mortality accurately with AUC of 0.88 (95% CI 0.87-0.88).

The magnitude and direction of individual feature contribution to prediction are inferred from the summary plot of Shapley values sorted by the descending order of feature impact (Multimedia Appendix 8); an increase in age [43-45], respiration rate [46], BUN [45,47,48], and aspartate transaminase [45,49], and a decrease in oxygen saturation [7,50], platelet count [26,51,52], and albumin [45,53] are associated with the increase in mortality risk.

### Comparison With Existing Benchmark

**Figure 5:**
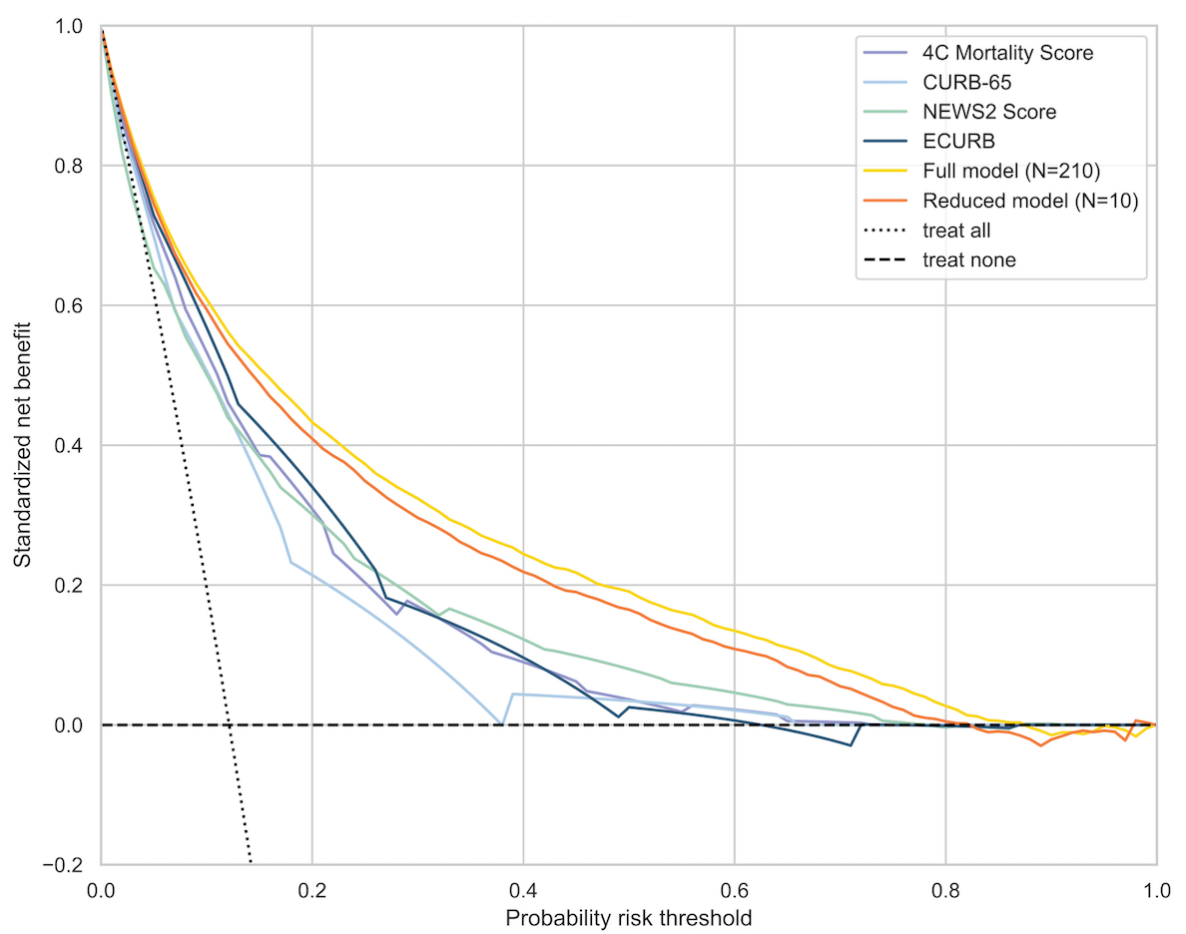
Decision curve analysis of standardized net benefit across different risk thresholds. Dotted line represents the scenario if everyone is treated; dashed line represents the scenario if none is treated.

The model shares commonalities (eg, age, respiration rate, blood pressures, pulse, BUN, SpO_2_, albumin) with existing prognostic scores for community-acquired pneumonia or COVID-19 [5,26,27]; however, with automated feature selection from comprehensive input covariates, and machine learning algorithm, it compares favorably with existing scores across diagnostic statistics (Table 3) and shows greater clinical utility across a wide range of probability thresholds (Figure 5) in decision curve analysis.

## Discussion

### Summary of Principal Findings

In this paper, we have adopted a systematic framework of developing and evaluating various machine learning techniques in predicting COVID-19 prognosis on near real-time, large-size EHR data in the United States. Boosting-based algorithms (XGBoost and LightGBM) have consistently outperformed other machine learning algorithms and COVID-19 benchmark risk scores with higher accuracy on the test data set (AUC 0.89, 95% CI 0.88-0.89) and on the prospective test data set (AUC 0.85, 95% CI 0.85-0.86), and better clinical utility on decision curve analysis. After further simplification of the model to only 10 clinical features, relative to full model it provides comparable discriminatory performance (AUC 0.88 95% CI 0.87-0.88) and clinical utility.

### Predictors

A major strength of this study is the use of near real-time, large-size EHR data, resulting in predictors that are highly representative and relevant to clinical practice. We have restricted the analysis to commonly measured covariates with less than 30% (15,211/50,703) of missing values among the cohort. A higher coverage cutoff precludes key predictors such as oxygen saturation [7,50], respiration rate [46], and BUN [47,48] leading to degradation of model performance (Multimedia Appendices 9 and 10).

Postadmission treatment is not a major predictor of model performance; they are not included in the final analysis, which results in minimal impact of the model performance on all-cause mortality. Patients are presented at different disease trajectories when admitted to hospital, with some being in critical condition; for instance, among 9564 ICU patients, 4745 (49.61%) were admitted to ICU on day 1 of hospital admission. The relationship between treatment type and outcome is therefore confounded by the stages of the disease course.

Age is identified as a crucial predictor for adverse outcomes [44]. It increases almost monotonically with health outcomes such as mortality and ARDS, but nonmonotonically with resource-dependent outcomes, such as ICU admission and invasive mechanical ventilation/ECMO, as these outcomes are closely associated with the availability of health care resources such as ventilator and ICU rooms. This is more noticeable for elderly patients over 75 years, who are disadvantaged for mechanical ventilation and ICU, presumably due to the scarcity of health care resources during the pandemic, though they are at highest mortality risk (Multimedia Appendix 11).

### Clinical Application

When applying the model to clinical setting, threshold selection is of great practical importance in producing dichotomous predictions. In the data-driven, cost-agnostic approach, threshold is derived numerically from the AUC curve (Figure 3), which maximizes the Youden index [34] (*P*=.13). When the model is applied to inform clinical decision making, such as identifying patients for dexamethasone treatment, insights from relevant clinical trials could guide threshold calculation. For instance, the findings from the Randomised Evaluation of COVID-19 Therapy (RECOVERY) trial [54], a large-enrollment, randomized controlled trial of dexamethasone, indicate a mortality risk reduction of 4.84% among patients who received oxygen therapy or were mechanically ventilated (393/1603, 24.52%) compared with the control group (965/3287, 29.36%); conversely, there was an increase in mortality risk of 3.74% among patients who require no oxygen (dexamethasone group [89/501, 17.76%] vs usual care group [145/1034, 14.02%]). When the model is applied clinically as a prognostic tool to identify patients who will receive dexamethasone, cost of false negatives (ie, misclassifying patients as low risk, and therefore they missed dexamethasone treatment) is 4.84%, and cost of false positive (ie, misclassifying patients as high risk) is 3.74% when cost of misclassification is expressed as an increase in mortality risk. Given the mortality rate of 1592/6425 (24.78%) from the RECOVERY trial [54], the threshold is found from the AUC curve at *P*=.33, where the slope of curve [33,37] is (0.0374/0.0484) x (1 – 0.248)/(0.248) = 2.35. Model performances are evaluated at these 2 thresholds in Table 3.

**Table 3:** Comparison with existing risk scores evaluated on test data sets to predict 28-day all-cause mortality. Sensitivity and specificity were evaluated at 2 different thresholds.

Risk score	AUC^a^ (95% CI), %	Threshold 1^b^		Threshold 2^c^		n^d^
		Sensitivity, %	Specificity, %	Sensitivity, %	Specificity, %	
Acute Physiology and Chronic Health Evaluation II	72.3 (69.5-74.9)	66.2	68.5	92.4	26.0	1769
Respiratory Rate-Oxygenation Index	68.5 (67.0-70.0)	28.2	92.7	54.2	78.3	16,640
CURB-65	78.7 (77.6-79.7)	36.2	92.4	77.2	69.1	15,001
E-CURB	81.9 (80.3-83.3)	63.4	83.4	87.3	61.3	5772
National Early Warning Score 2 score	82.9 (81.7-84.2)	51.6	91.2	75.0	77.0	14,112
Coronavirus Clinical Characterization Consortium Mortality score	82.2 (80.7-83.5)	62.3	83.8	71.8	75.7	6979
Baseline model	73.8 (73.2-74.5)	44.8	83.4	80.2	54.9	25,615
Full model	89.2 (88.1-90.3)	63.1	92.2	85.2	76.4	8493
N10 model	88.9 (88.0-90.0)	65.9	90.9	81.4	79.3	10,688

^a^AUC: area under the receiver operating characteristic curve.

^b^Threshold 1 is a clinically relevant threshold that identifies patients for dexamethasone treatment; costs of FP and FN are expressed in terms of mortality risk.

^c^Threshold 2 is derived from a cost-agnostic approach and is located at the point on the area under the receiver operating characteristic curve that maximizes the Youden index.

^d^Number of hospitalized patients in the test data set and the postdevelopment test data set with complete case.

These 2 thresholds (*P*=.13 and .24) are similar to the intermediate- and high-risk cutoffs used to define the severity of pneumonia [1,55,56]. Based on these approaches, we derived 2 clinically meaningful thresholds (Table 4), stratifying patients into (1) low-to-intermediate risk (*P*≤.13, observed mortality rate = 315/8065, 3.91%); (2) high risk (0.13<*P*≤.24, observed mortality rate = 225/1170, 19.23%); and (3) very high risk (*P*>.24, observed mortality rate = 787/1517, 51.88%). Scenario-based threshold can be substituted with appropriate clinical trial insights according to different treatment options.

**Table 4:** Mortality rate comparison across different risk groups on the test and postdevelopment prospective test data sets. Three risk groups were defined as (1) low-to-intermediate–risk group (*P*≤.13), (2) high risk (.13<*P*≤.24), and (3) very high risk (*P*>.24). The threshold probabilities are obtained from receiver operating characteristic analysis, which (1) maximizes the Youden index (*P*=.13), or (2) defined by clinical utility of dexamethasone (*P*=.24) from the RECOVERY^a^ trial.

Risk group	Test data set		Prospective test data set	
	Patients, n (%) (n=10,752)	Deaths, n (%) (n=1327)	Patients, n (%) (n=14,863)	Deaths, n (%) (n=1782)
Low–intermediate	8065 (75.01)	315 (3.91)	11,049 (74.34)	512 (4.63)
High	1170 (10.88)	225 (19.23)	1743 (11.73)	327 (18.76)
Very high	1517 (14.11)	787 (51.88)	2071 (13.93)	943 (45.53)

^a^RECOVERY: Randomised Evaluation of COVID-19 Therapy.

### Strengths

The strengths of this research include the large size of data set, longitudinal nature, and near real-time update of the data release. The Optum database provides patient-level information with a diverse mix of geographic regions, insurance types, socioeconomic status, and ethnicity. A comprehensive list of 386 input covariates from baseline and at admission was included in the analysis based on epidemiological and clinical characteristics of COVID-19 cases; the end-to-end pipeline automates feature selection and model development process, producing risk factors that are both commonly measured at admission with wide coverage among study cohort and concordant with similar risk scores. This helps to improve the usability of the model without extensive electronic medical record integration or feeding the model with continuous data streams. The systematic approach and rigorous validations demonstrate consistent model performance to predict even beyond the period of data collection, with satisfactory discriminatory power and great clinical utility. Overall, the study offers an accurate, validated, and reliable prediction model based on only 10 clinical features as a prognostic tool for stratifying patients with COVID-19 into intermediate-, high-, and very high-risk groups. We envision this model to be used on the day of hospital admission at an inpatient setting where resource triaging is most relevant and early identification of high-risk patient is the key.

### Limitations

There are several limitations in our study. First, the Optum COVID-19 database, being an EHR database, may not capture patients’ entire interaction with health care systems because patients can switch between different hospitals or health care systems. This impacts several aspects of the study, from assessment of baseline comorbidity and comedication, to capture of outcomes during follow-up. Although we have identified a minimum of 10-week period from database refresh date to COVID-19 diagnosis date to allow for capture of follow-up data and outcomes, it is possible additional data lag is still present, challenging the completeness and accuracy of outcome assessment.

Because of Health Insurance Portability and Accountability Act (HIPAA)–compliance protection, patients over 89 years were included as a single category of age in the data set, with age being an important risk predictor of mortality. This can potentially lead to some performance degradation for patients aged over 89 years. Additional data, such as symptoms since onset, could aid in early prediction of aggressive COVID-19 progression, but these were available for less than 70% (35,493/50,703) of patients in the data set; if we had adequate data on patient symptoms and the use of oxygen therapies, model performance would likely improve. Similarly, this negatively impacts the evaluation of existing prognostic scores that require FiO_2_. We have referred to the best currently available information on clinical trial for threshold calculation, and there could still exist differences in patient population between the RECOVERY trial and this work. Additional work is required for validating the results on vaccinated population.

### Conclusions

In this study, we presented a systematic framework of model development based on a variety of machine learning techniques, combined with rigorous validation on statistically meaningful sample size. The model demonstrates consistent performance to predict even beyond the period of data collection. The parsimonious model with only 10 clinical features (age, systolic and diastolic blood pressures, respiration rate, pulse, temperature, BUN, SpO_2_, albumin, and presence of major cognitive disorder) offers an accurate, validated, and calibrated prediction to stratifying COVID-19 patients into intermediate-, high-, and very high-risk groups. This simple predictive tool is shared with a wider health care community (Multimedia Appendix 12), to enable service as an early warning system to alert physicians of possible high-risk patients.

## Appendix

Multimedia Appendix 1 Model input and variable transformation. In the preliminary analysis, a total of 386 covariates with <30% missingness are incorporated as model input.

Multimedia Appendix 2 Summary of missingness of vital and lab variables among the study cohort.

Multimedia Appendix 3 Sensitivity analysis on model performances (AUC, 95% CI) between study cohort (n=50,703 in orange) and lab confirmed cohort (n=38,277 in blue) which is a subset of study cohort; (a) model performances on test dataset; (b) on post-development prospective test dataset.

Multimedia Appendix 4 Model performance (AUC) during preliminary analysis (top: test dataset (N=10,752); bottom: post-development prospective test dataset (N=14,863). In the preliminary analysis, a total of 386 covariates (with <30% missingness) are incorporated as model input.

Multimedia Appendix 5 Calibration curve during preliminary analysis (top panel: test dataset (N=10,752); bottom panel: post-development prospective test dataset (N=14,863)). In the preliminary analysis, a total of 386 covariates (with <30% missingness) are incorporated as model input.

Multimedia Appendix 6 Runtime comparison among 6 candidate algorithms.

Multimedia Appendix 7 Frequency heatmap of top 20 features at 5-variable to 20-variable algorithms (step size = 1). (a) all-cause mortality; (b) mechanical ventilation including ECMO; (c) ARDS including respiratory failure; (d) ICU admission during final analysis, excluding covariates of post-admission treatment.

Multimedia Appendix 8 SHAP summary plot on 28-day mortality on aggregated datasets (test dataset and post-development prospective test dataset).

Multimedia Appendix 9 Sensitivity analysis on model performances (AUC) between non-imputed (in orange) and imputed data (in blue); (a) 28-day mortality; (b) 28-day ICU admission; (c) composite of 28-day ARDS and respiratory failure; (d) composite of 28-day ECMO and invasive ventilation usage. Left – test dataset; right – post-development prospective test dataset.

Multimedia Appendix 10 Sensitivity analysis on model performances (AUC) on non-imputed dataset with different thresholds of covariate coverage (10%, 30%, 50%, 70%, 80%, 90%) among the study cohort; (a) all-cause mortality; (b) ICU admission; (c) respiratory failure including ARDS; (d) invasive mechanical ventilation including ECMO. Left panel – test dataset; right panel – post-development prospective test dataset.

Multimedia Appendix 11 SHAP dependence plot between age and four outcomes (top left: 28-day mortality; top right: composite of 28-day ARDS and respiratory failure; bottom left: 28-day ICU admission; bottom right: 28-day ECMO or ventilator. The features are colored by (a) minimum SpO_2_ on admission; (b) respiration rate; (c) lymphocyte count; (d) BUN.

Multimedia Appendix 12 Gitlab repository.
