# Supplementary material for: The Development and Validation of Simplified Machine Learning Algorithms to Predict Prognosis of Hospitalized Patients With COVID-19: Multicenter, Retrospective Study
Source: J Med Internet Res. 2022 Jan 21;24(1):e31549. doi: 10.2196/31549 (PMC8785956; doi:10.2196/31549)
Supplement: Multimedia Appendix 1 [file jmir_v24i1e31549_app1.pdf]

Multimedia Appendix 1. Model input and variable transformation. In the preliminary analysis, a total of 386 covariates with < 30% missingness are incorporated as model input.

Covariate	Unit	Window	Examples	Transformation
Patient demographic				
Age	years	Latest	Numerical values[a]	-
Sex	-	Latest	• Male<br>• Female	Convert to binary variables
CENSUS division	-	Latest	• East North Central<br>• East South Central<br>• Middle Atlantic<br>• Mountain<br>• New England<br>• Pacific<br>• South Atlantic/West South Central<br>• West North Central<br>• Other/Unknown	Convert categorical to dummy variables
Ethnicity	-	Latest	• Hispanic<br>• Not Hispanic<br>• Unknown	Convert categorical to dummy variables
Race	-	Latest	• African American<br>• Asian<br>• Caucasian<br>• Other/Unknown	Convert categorical to dummy variables
Insurance	-	Latest	• Commercial<br>• Medicare<br>• Medicaid<br>• Other payor type<br>• Uninsured<br>• Unknown	Convert categorical to dummy variables
Lifestyle factors				
Smoke	-	Latest	• Current<br>• Previous<br>• Never<br>• Unknown	Convert categorical to dummy variable
BMI	kg/m^2	Latest	Numerical values[a]	-
COVID-19 ascertainment				
Diagnosis month	-	-	Jan – Nov	Convert categorical to dummy variable
Diagnosis site	-	-	• Inpatient<br>• Other	Binary
Hospitalization lag	-	-	Number of days from COVID diagnosis or test date to admission date	Numerical

Comorbidity (*non-exhaustive list*)				
CAD	-	Baseline	Yes/No	Binary
Cancer	-	Baseline	Yes/No	Binary
CKD	-	Baseline	Yes/No	Binary
COPD	-	Baseline	Yes/No	Binary
Dementia	-	Baseline	Yes/No	Binary
Diabetes Mellitus	-	Baseline	Yes/No	Binary
Hypertension	-	Baseline	Yes/No	Binary
PAD	-	Baseline	Yes/No	Binary
Stroke	-	Baseline	Yes/No	Binary
Baseline medication (*non-exhaustive list*)				
Antibiotics	-	Baseline	Yes/No	Binary
Anticoagulants	-	Baseline	Yes/No	Binary
Antidiabetics	-	Baseline	Yes/No	Binary
Antihypertensives	-	Baseline	Yes/No	Binary
Antivirals	-	Baseline	Yes/No	Binary
Diuretics	-	Baseline	Yes/No	Binary
DMARDS	-	Baseline	Yes/No	Binary
Immunomodulators	-	Baseline	Yes/No	Binary
Steroids	-	Baseline	Yes/No	Binary
Vital and Observations				
Diastolic BP	mm Hg	Index date only[b]	Numerical values[a]	-
Systolic BP	mm Hg	Index date only[b]	Numerical values[a]	-
Heart rate	bpm	Index date only[b]	Numerical values[a]	-
Pulse	bpm	Index date only[b]	Numerical values[a]	-
Resp rate	/min	Index date only[b]	Numerical values[a]	-
Temperature	ºC	Index date only[b]	Numerical values[a]	-
Lab (*non-exhaustive list*)				
AST	IU/L	Index date only[c]	Numerical values[a]	-
BUN	mg/dL	Index date only[c]	Numerical values[a]	-

CRP	mg/L	Index date only[c]	Numerical values[a]	-
Lymphocyte	x 10^9/L	Index date only[c]	Numerical values[a]	-
SpO_2	%	Index date only[c]	Numerical values[a]	-
Platelet	x 10^9/L	Index date only[c]	Numerical values[a]	-
Minimum or first oxygen measurement				
SaO_2	%	Index date only[b]	Numerical values[a]	-
SpO_2	%	Index date only[b]	Numerical values[a]	-
Post-admission treatment (*non-exhaustive list*)[d]				
Antibiotics	-	Index date only	Yes/No	Binary
Anticoagulants	-	Index date only	Yes/No	Binary
Antidiabetics	-	Index date only	Yes/No	Binary
Antihypertensives	-	Index date only	Yes/No	Binary
Antivirals	-	Index date only	Yes/No	Binary
Diuretics	-	Index date only	Yes/No	Binary
DMARDS	-	Index date only	Yes/No	Binary
Immunomodulators	-	Index date only	Yes/No	Binary
Steroids	-	Index date only	Yes/No	Binary

[a]Numerical values are normalized by min-max standardization for logical regression models (Lasso or Ridge regularized), otherwise no transformation was applied to tree-based models (XGBoost, Random forest and Decision trees).
[b]The first and the minimum measurement on the index date were selected.
[c]If there are multiple measurements on the index date, a single measurement was randomly selected.
[d]Excluded from the final analysis.
