# Supplementary material for: The Development and Validation of Simplified Machine Learning Algorithms to Predict Prognosis of Hospitalized Patients With COVID-19: Multicenter, Retrospective Study
Source: J Med Internet Res. 2022 Jan 21;24(1):e31549. doi: 10.2196/31549 (PMC8785956; doi:10.2196/31549)
Supplement: Multimedia Appendix 2 [file jmir_v24i1e31549_app2.pdf]

Multimedia Appendix 2. Summary of missingness of vital and lab variables among the study cohort.

Vital*	Missing value (%)	Comments
Respiratory rate (breaths/min)	24.3%	Included as model input
Systolic blood pressure, SBP (mm Hg)	14.0%	Included as model input
Diastolic blood pressure, DBP (mm Hg)	13.4%	Included as model input
Temperature (ºC)	13.3%	Included as model input
Heart rate (bpm)	72.4%	Not included as input to the model, missingness ≥ 30%
Pulse (bpm)	24.3%	Included as model input
**Lab**	**Missing value (%)**	**Comments**
Alkaline phosphatase, ALP (IU/L)	25.6%	Included as model input
Alanine aminotransferase, ALT (IU/L)	25.4%	Included as model input
Aspartate aminotransferase, AST (IU/L)	26.2%	Included as model input
Albumin (g/dL)	25.6%	Included as model input
Amylase (IU/L)	99.3%	Not included as input to the model, missingness ≥ 30%
Anion gap (mEq/L)	15.5%	Included as model input
BNP (pg/mL)	75.7%	Not included as input to the model, missingness ≥ 30%
BUN (mg/dL)	12.1%	Included as model input
Bicarbonate (mmol/L)	11.9%	Included as model input
Bilirubin, total (mg/dL)	25.5%	Included as model input
Bilirubin, direct (mg/dL)	75.9%	Not included as input to the model, missingness ≥ 30%
C reactive protein, CRP (mg/dL)	58.2%	Not included as input to the model, missingness ≥ 30%
CRP, high sensitivity (mg/dL)	97.4%	Not included as input to the model, missingness ≥ 30%
Chloride (mmol/L)	14.1%	Included as model input
Creatinine (mg/dL)	14.1%	Included as model input
D-Dimer (ng/mL)	62.3%	Not included as input to the model, missingness ≥ 30%
Erythrocyte sedimentation rate (mm/hr)	94.2%	Not included as input to the model, missingness ≥ 30%
Ferritin (ng/mL)	61.2%	Not included as input to the model, missingness ≥ 30%
Fibrinogen (mg/dL)	86.7%	Not included as input to the model, missingness ≥ 30%
γ-Glutamyltransferase, GGT (IU/L)	97.4%	Not included as input to the model, missingness ≥ 30%
Globulin (g/dL)	81.2%	Not included as input to the model, missingness ≥ 30%
Glucose (mg/dL)	10.4%	Included as model input
HDL (mg/dL)	98.3%	Not included as input to the model, missingness ≥ 30%
HbA1c (%)	92.2%	Not included as input to the model, missingness ≥ 30%
Hemoglobin (g/dL)	11.7%	Included as model input
Lactate dehydrogenase (U/L)	61.1%	Not included as input to the model, missingness ≥ 30%
LDL (mg/dL)	98.3%	Not included as input to the model, missingness ≥ 30%
Lipase (U/L)	86.1%	Not included as input to the model, missingness ≥ 30%
Lymphocyte count ($\times 10^9$/L)	15.9%	Included as model input
Lymphocyte (%)	17.8%	Included as model input
Monocyte count ($\times 10^9$/L)	18.3%	Included as model input
Monocyte (%)	17.8%	Included as model input
NT-proBNP (pg/mL)	89.8%	Not included as input to the model, missingness ≥ 30%
Neutrophil count ($\times 10^9$/L)	16.1%	Included as model input
Neutrophil (%)	17.6%	Included as model input
PT (s)	60.6%	Not included as input to the model, missingness ≥ 30%
PTT (s)	68.1%	Not included as input to the model, missingness ≥ 30%
$PaCO_2$ (mm Hg)	86.2%	Not included as input to the model, missingness ≥ 30%
PaO_2 (mm Hg)	86.3%	Not included as input to the model, missingness ≥ 30%
Platelet count ($\times 10^9$/L)	12.1%	Included as model input
Potassium (mmol/L)	12.1%	Included as model input
Procalcitonin (ng/mL)	64.8%	Not included as input to the model, missingness ≥ 30%
Protein, total (g/dL)	27.5%	Included as model input
RDW-CV (%)	12.4%	Included as model input
SaO_2 (%)	89.2%	Not included as input to the model, missingness ≥ 30%
SaO_2 (%)*	90.7%	Not included as input to the model, missingness ≥ 30%
SaO_2 (%)^	89.0%	Not included as input to the model, missingness ≥ 30%
Sodium (mmol/L)	11.8%	Included as model input
SpO_2 (%)	11.0%	Included as model input
SpO_2 (%)*	12.0%	Included as model input
SpO_2 (%)^	11.1%	Included as model input

Triglyceride (mg/dL)	93.6%	Not included as input to the model, missingness ≥ 30%
Total Cholesterol (mg/dL)	98.3%	Not included as input to the model, missingness ≥ 30%
Vitamin D (ng/mL)	99.4%	Not included as input to the model, missingness ≥ 30%
White blood cell count ($\times 10^9$/L)	12.0%	Included as model input
Estimated GFR (ml/min/1.73m^2)	92.6%	Not included as input to the model, missingness ≥ 30%
pH, arterial blood	86.1%	Not included as input to the model, missingness ≥ 30%

*first measurement after hospital admission
^minimum measurement after hospital admission
