# Supplementary material for: The Development and Validation of Simplified Machine Learning Algorithms to Predict Prognosis of Hospitalized Patients With COVID-19: Multicenter, Retrospective Study
Source: J Med Internet Res. 2022 Jan 21;24(1):e31549. doi: 10.2196/31549 (PMC8785956; doi:10.2196/31549)
Supplement: Multimedia Appendix 3 [file jmir_v24i1e31549_app3.pdf]

**Multimedia Appendix 3. Sensitivity analysis on model performances (AUC, 95% CI) between study cohort (N=50,703 in orange) and lab confirmed cohort (N=38,277 in blue) which is a subset of study cohort; (a) model performances on test dataset; (b) on post-development prospective test dataset.**

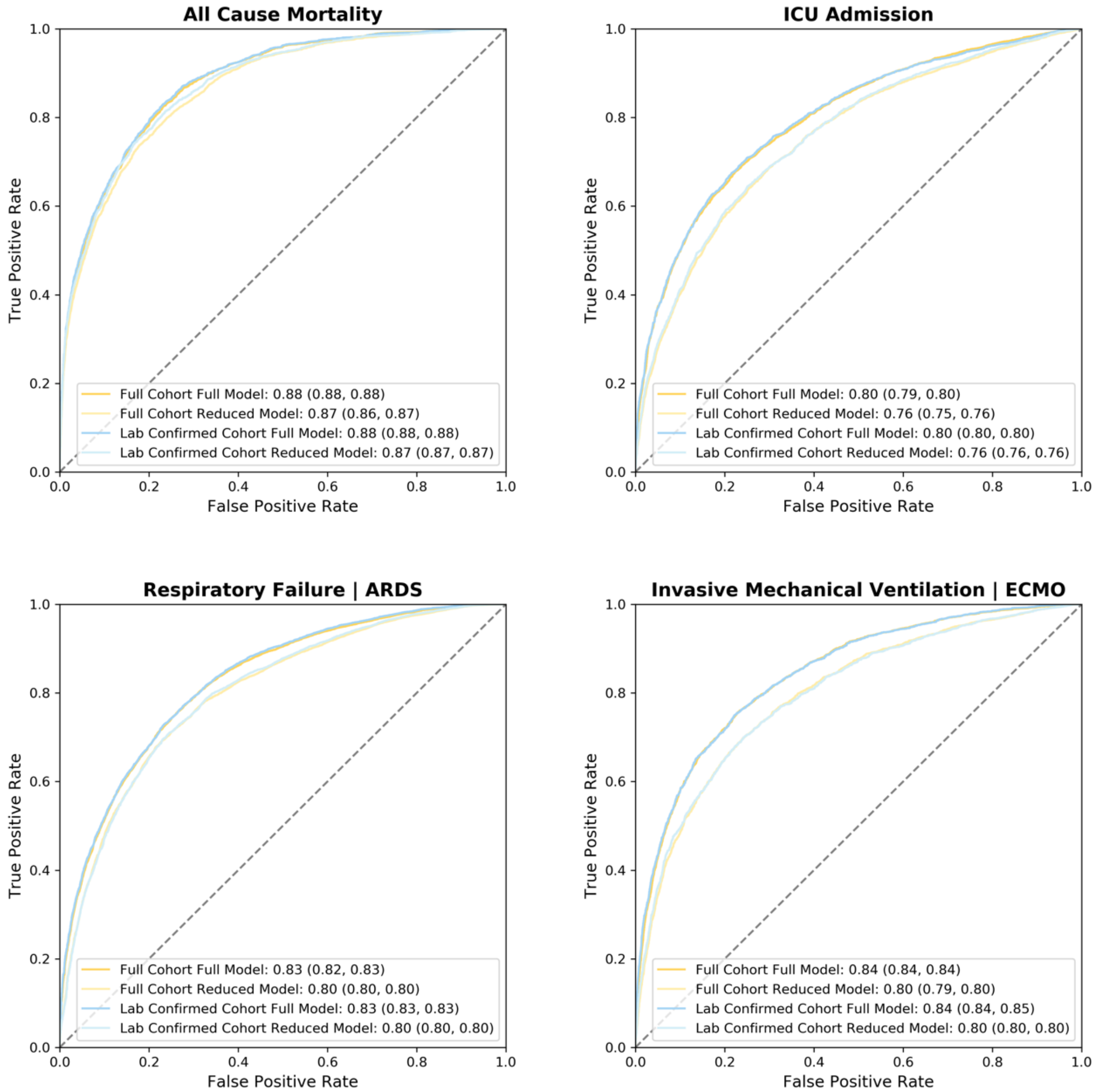


(a)

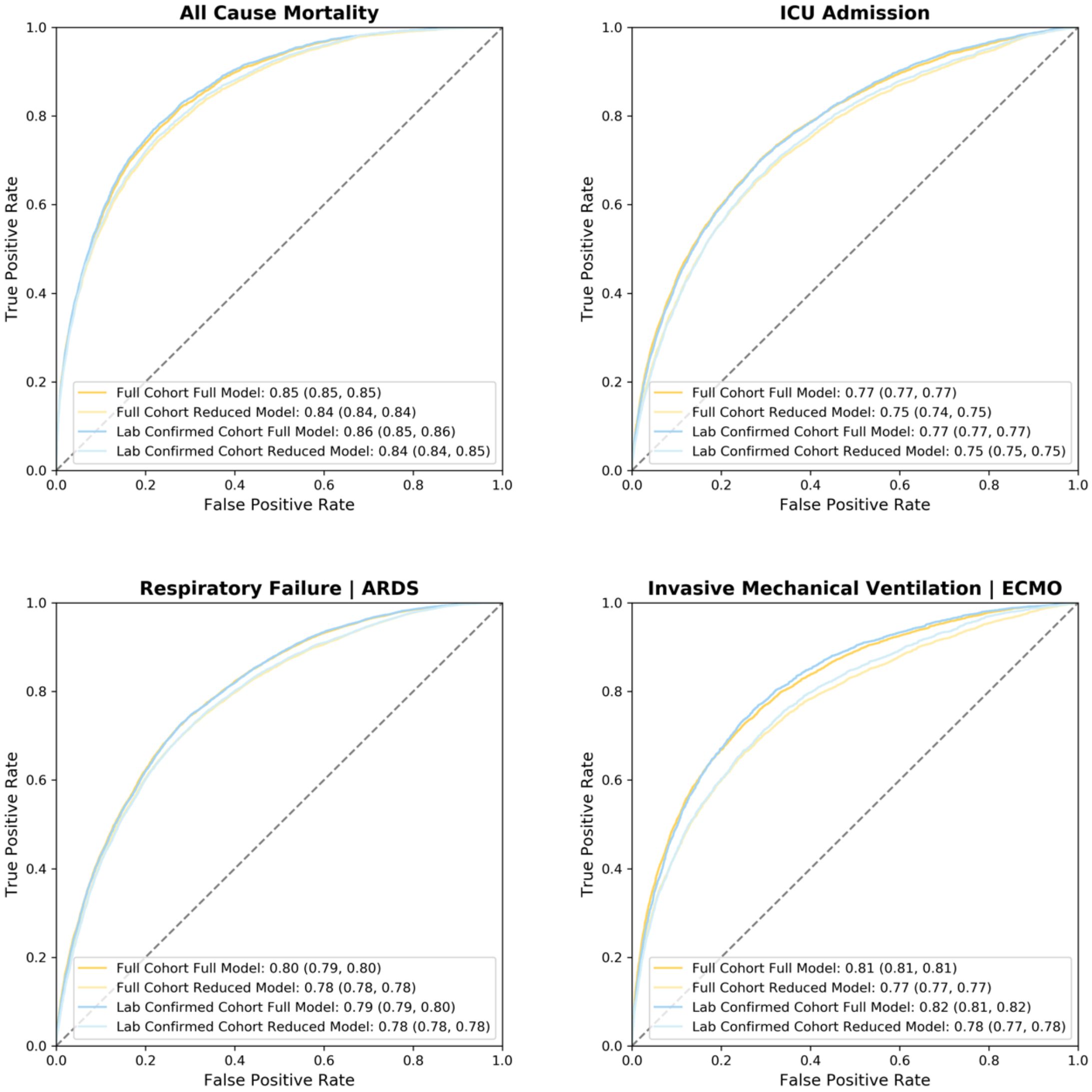

All Cause Mortality
True Positive Rate
False Positive Rate
Full Cohort Full Model: 0.85 (0.85, 0.85)
Full Cohort Reduced Model: 0.84 (0.84, 0.84)
Lab Confirmed Cohort Full Model: 0.86 (0.85, 0.86)
Lab Confirmed Cohort Reduced Model: 0.84 (0.84, 0.85)
ICU Admission
True Positive Rate
False Positive Rate
Full Cohort Full Model: 0.77 (0.77, 0.77)
Full Cohort Reduced Model: 0.75 (0.74, 0.75)
Lab Confirmed Cohort Full Model: 0.77 (0.77, 0.77)
Lab Confirmed Cohort Reduced Model: 0.75 (0.75, 0.75)
Respiratory Failure | ARDS
True Positive Rate
False Positive Rate
Full Cohort Full Model: 0.80 (0.79, 0.80)
Full Cohort Reduced Model: 0.78 (0.78, 0.78)
Lab Confirmed Cohort Full Model: 0.79 (0.79, 0.80)
Lab Confirmed Cohort Reduced Model: 0.78 (0.78, 0.78)
Invasive Mechanical Ventilation | ECMO
True Positive Rate
False Positive Rate
Full Cohort Full Model: 0.81 (0.81, 0.81)
Full Cohort Reduced Model: 0.77 (0.77, 0.77)
Lab Confirmed Cohort Full Model: 0.82 (0.81, 0.82)
Lab Confirmed Cohort Reduced Model: 0.78 (0.77, 0.78)

(b)
