# Supplementary material for: The Development and Validation of Simplified Machine Learning Algorithms to Predict Prognosis of Hospitalized Patients With COVID-19: Multicenter, Retrospective Study
Source: J Med Internet Res. 2022 Jan 21;24(1):e31549. doi: 10.2196/31549 (PMC8785956; doi:10.2196/31549)

Multimedia Appendix 4. Model performance (AUC) during preliminary analysis (top: test dataset (N=10,752); bottom: post-development prospective test dataset (N=14,863)). In the preliminary analysis, a total of 386 covariates (with < 30% missingness) are incorporated as model input.

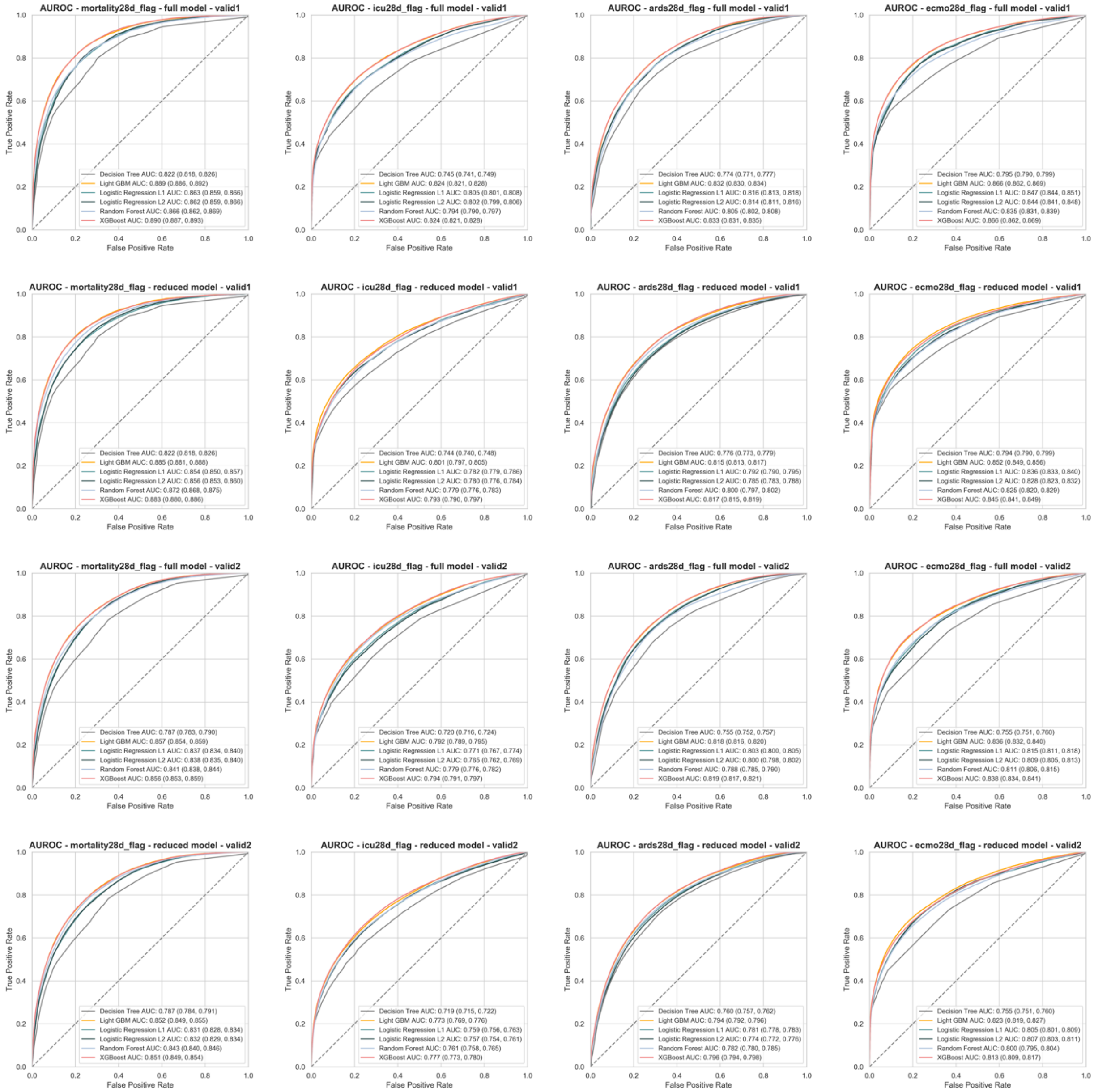

Supplement: Multimedia Appendix 4 [file jmir_v24i1e31549_app4.pdf]
