# Supplementary figures and images for: The Development and Validation of Simplified Machine Learning Algorithms to Predict Prognosis of Hospitalized Patients With COVID-19: Multicenter, Retrospective Study
Source: J Med Internet Res. 2022 Jan 21;24(1):e31549. doi: 10.2196/31549 (PMC8785956; doi:10.2196/31549)

**Multimedia Appendix 6. Runtime comparison among six candidate algorithms.**

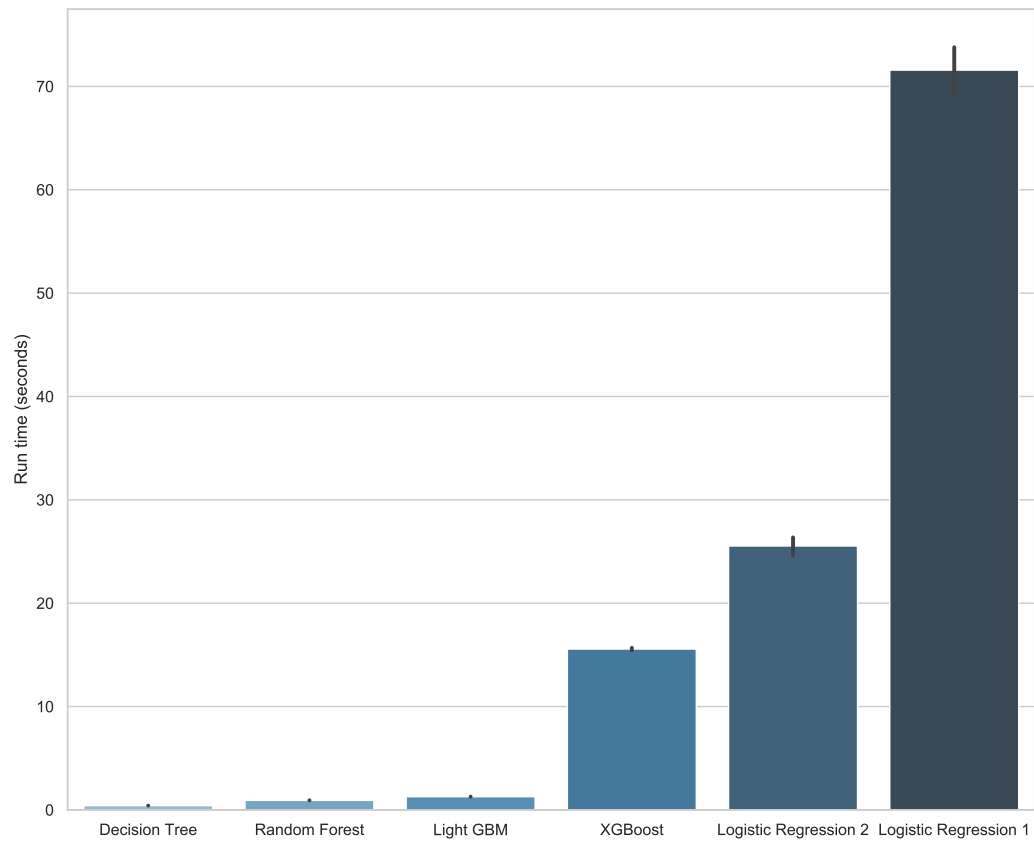

Supplement: Multimedia Appendix 6 [file jmir_v24i1e31549_app6.pdf]
