# Supplementary material for: The Development and Validation of Simplified Machine Learning Algorithms to Predict Prognosis of Hospitalized Patients With COVID-19: Multicenter, Retrospective Study
Source: J Med Internet Res. 2022 Jan 21;24(1):e31549. doi: 10.2196/31549 (PMC8785956; doi:10.2196/31549)
Supplement: Multimedia Appendix 7 [file jmir_v24i1e31549_app7.pdf]

Multimedia Appendix 7. Frequency heatmap of top 20 features at 5-variable to 20-variable algorithms (step size = 1). (a) all-cause mortality; (b) mechanical ventilation including ECMO; (c) ARDS including respiratory failure; (d) ICU admission during final analysis, excluding covariates of post-admission treatment.

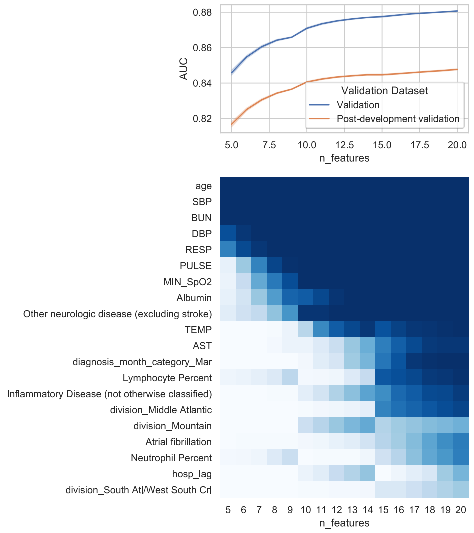

(a)

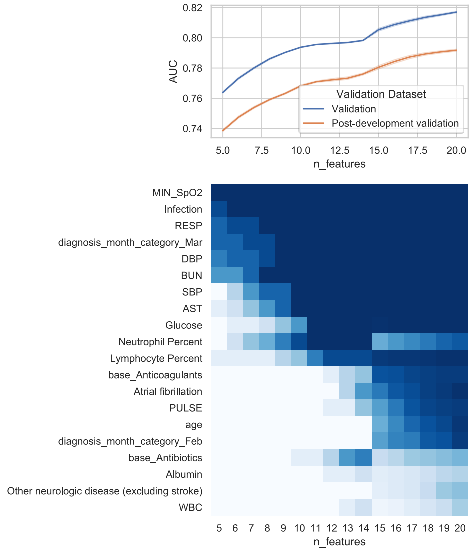

(b)

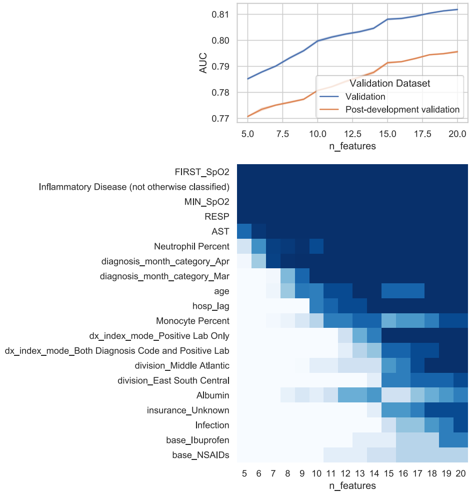

(c)

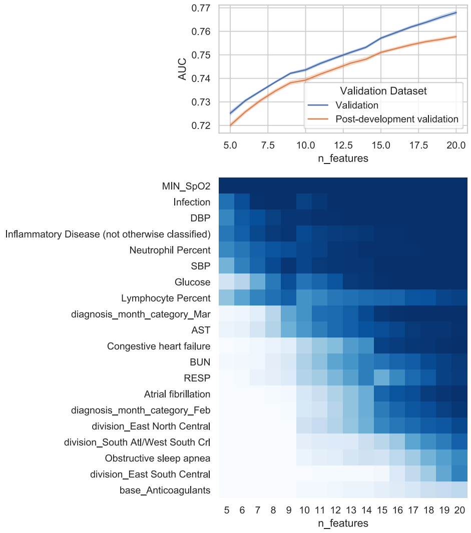

(d)
