# Supplementary material for: The Development and Validation of Simplified Machine Learning Algorithms to Predict Prognosis of Hospitalized Patients With COVID-19: Multicenter, Retrospective Study
Source: J Med Internet Res. 2022 Jan 21;24(1):e31549. doi: 10.2196/31549 (PMC8785956; doi:10.2196/31549)

Multimedia Appendix 8. SHAP summary plot on 28-day mortality on aggregated datasets (test dataset and post-development prospective test dataset).

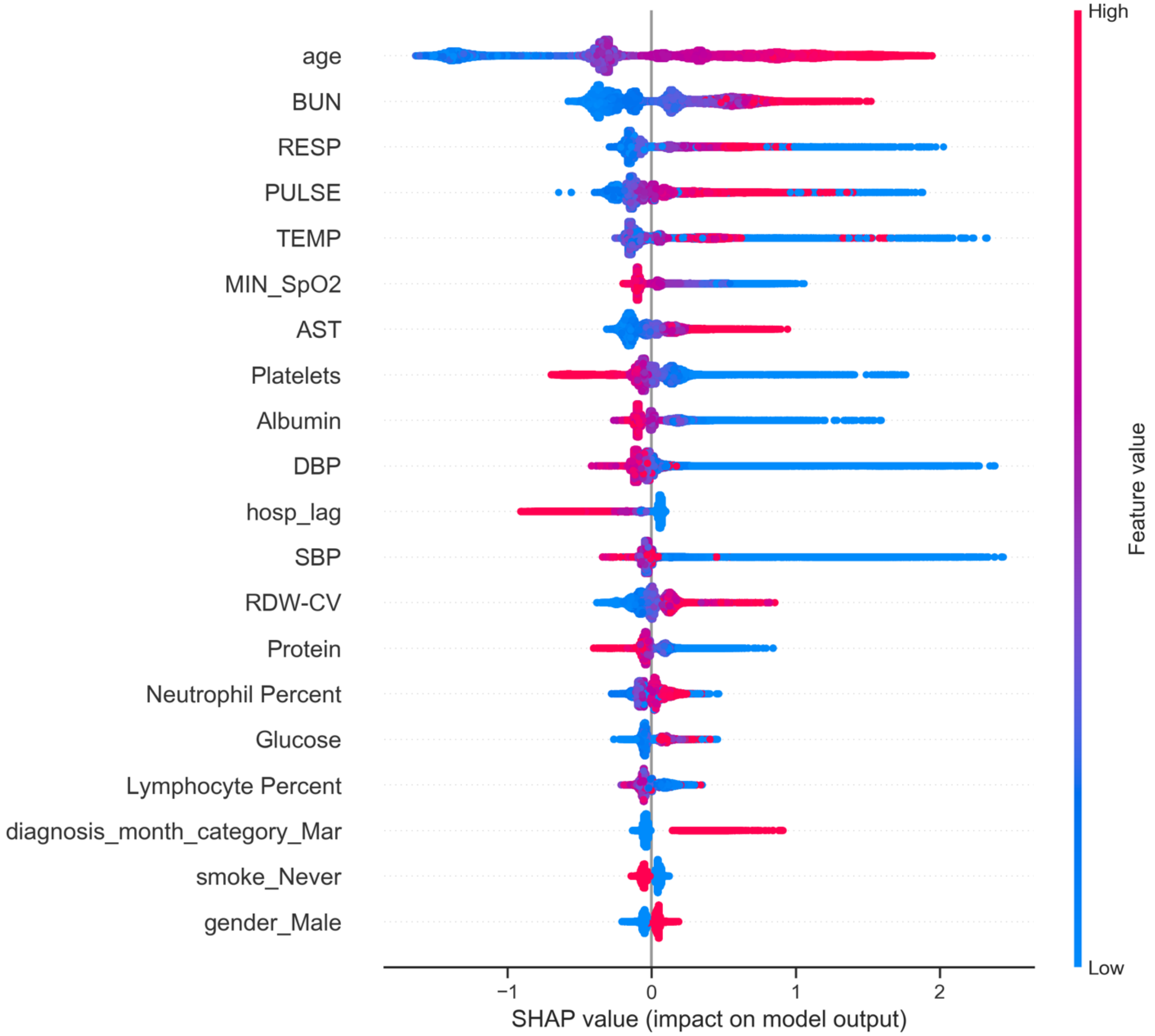

Supplement: Multimedia Appendix 8 [file jmir_v24i1e31549_app8.pdf]
