# Supplementary material for: The Development and Validation of Simplified Machine Learning Algorithms to Predict Prognosis of Hospitalized Patients With COVID-19: Multicenter, Retrospective Study
Source: J Med Internet Res. 2022 Jan 21;24(1):e31549. doi: 10.2196/31549 (PMC8785956; doi:10.2196/31549)
Supplement: Multimedia Appendix 9 [file jmir_v24i1e31549_app9.pdf]

Multimedia Appendix 9. Sensitivity analysis on model performances (AUC) between non-imputed (in orange) and imputed data (in blue); (a) 28-day mortality; (b) 28-day ICU admission; (c) composite of 28-day ARDS and respiratory failure; (d) composite of 28-day ECMO and invasive ventilation usage. Left – test dataset; right – post-development prospective test dataset.

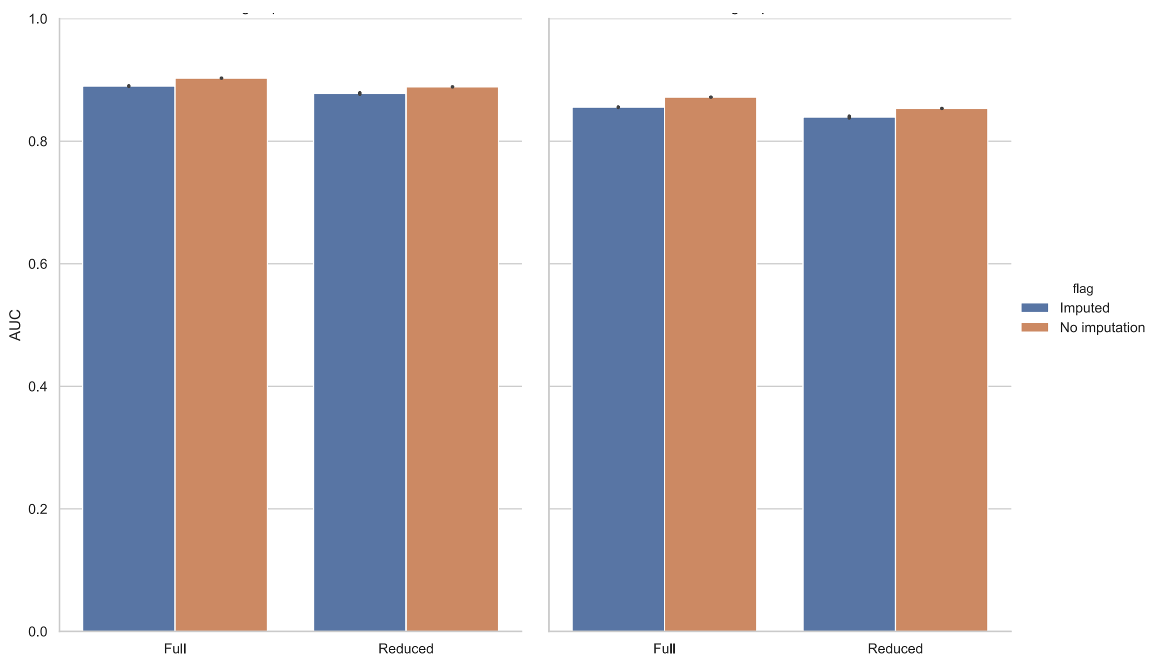

(a)

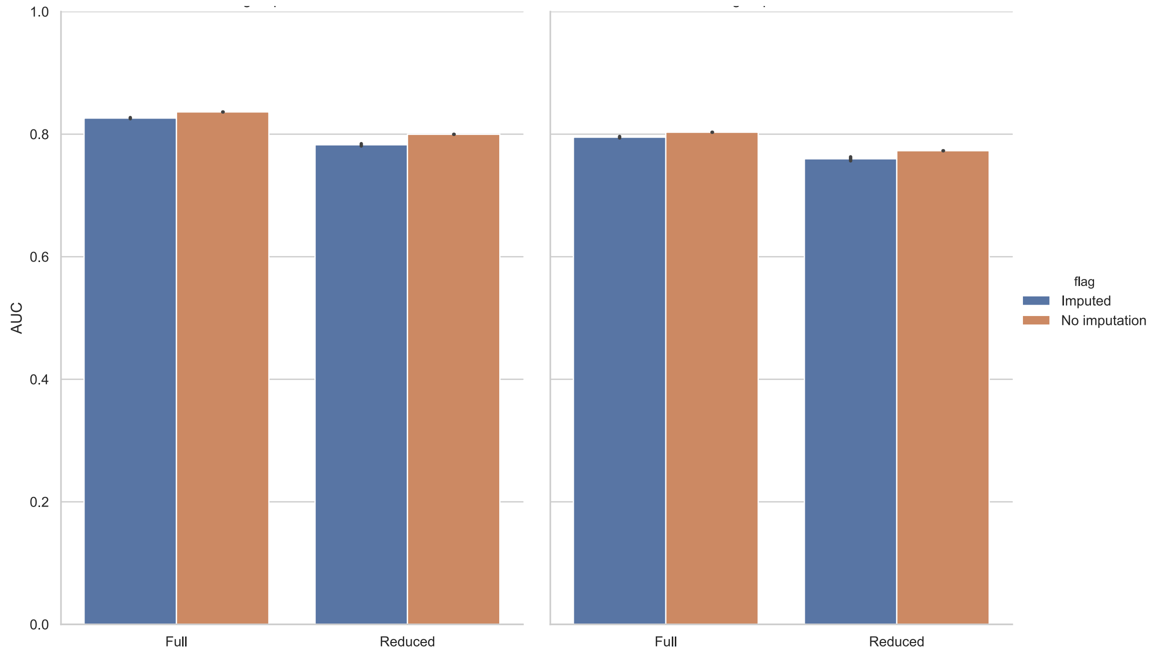

(b)

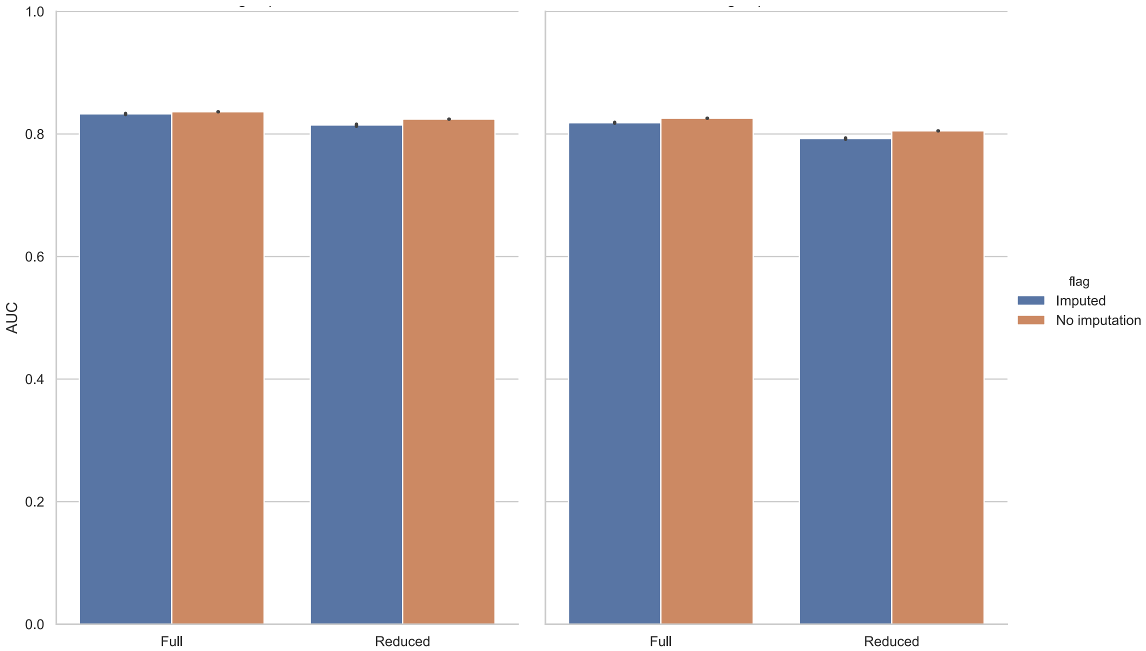
1.0
0.8
0.6
0.4
0.2
0.0
AUC
Full
Reduced
Full
Reduced
flag
Imputed
No imputation

(c)

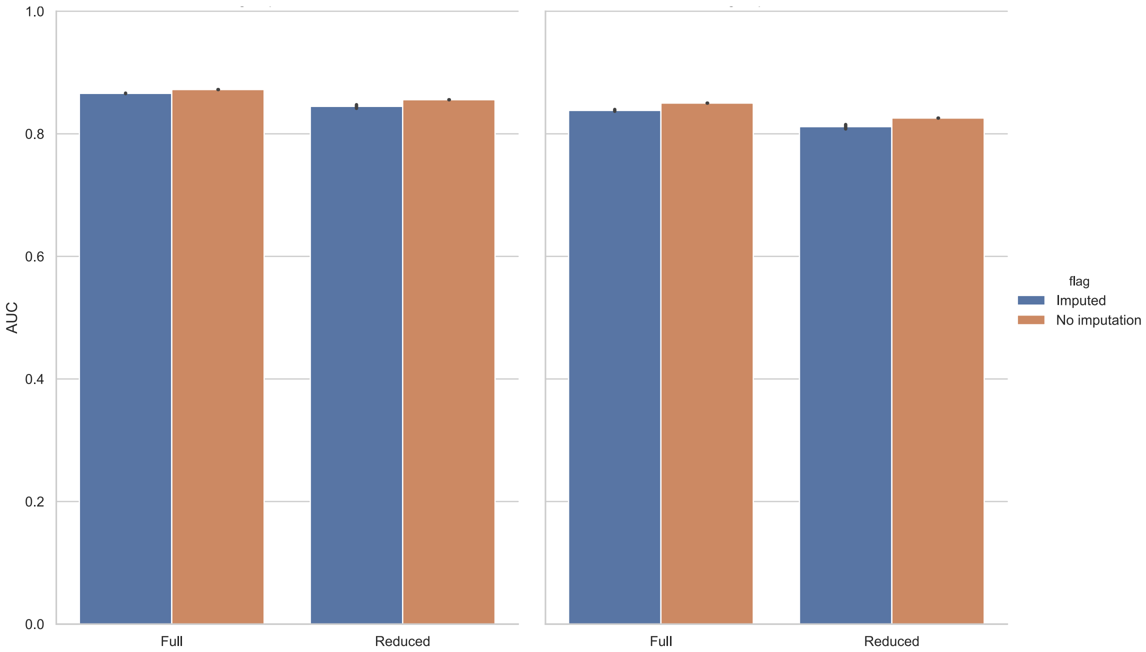
1.0
0.8
0.6
0.4
0.2
0.0
AUC
Full
Reduced
Full
Reduced
flag
Imputed
No imputation

(d)
