# Supplementary material for: The Development and Validation of Simplified Machine Learning Algorithms to Predict Prognosis of Hospitalized Patients With COVID-19: Multicenter, Retrospective Study
Source: J Med Internet Res. 2022 Jan 21;24(1):e31549. doi: 10.2196/31549 (PMC8785956; doi:10.2196/31549)
Supplement: Multimedia Appendix 10 [file jmir_v24i1e31549_app10.pdf]

Multimedia Appendix 10. Sensitivity analysis on model performances (AUC) on non-imputed dataset with different thresholds of covariate coverage (10%, 30%, 50%, 70%, 80%, 90%) among the study cohort; (a) all-cause mortality; (b) ICU admission; (c) respiratory failure including ARDS; (d) invasive mechanical ventilation including ECMO. Left panel – test dataset; right panel – post-development prospective test dataset.

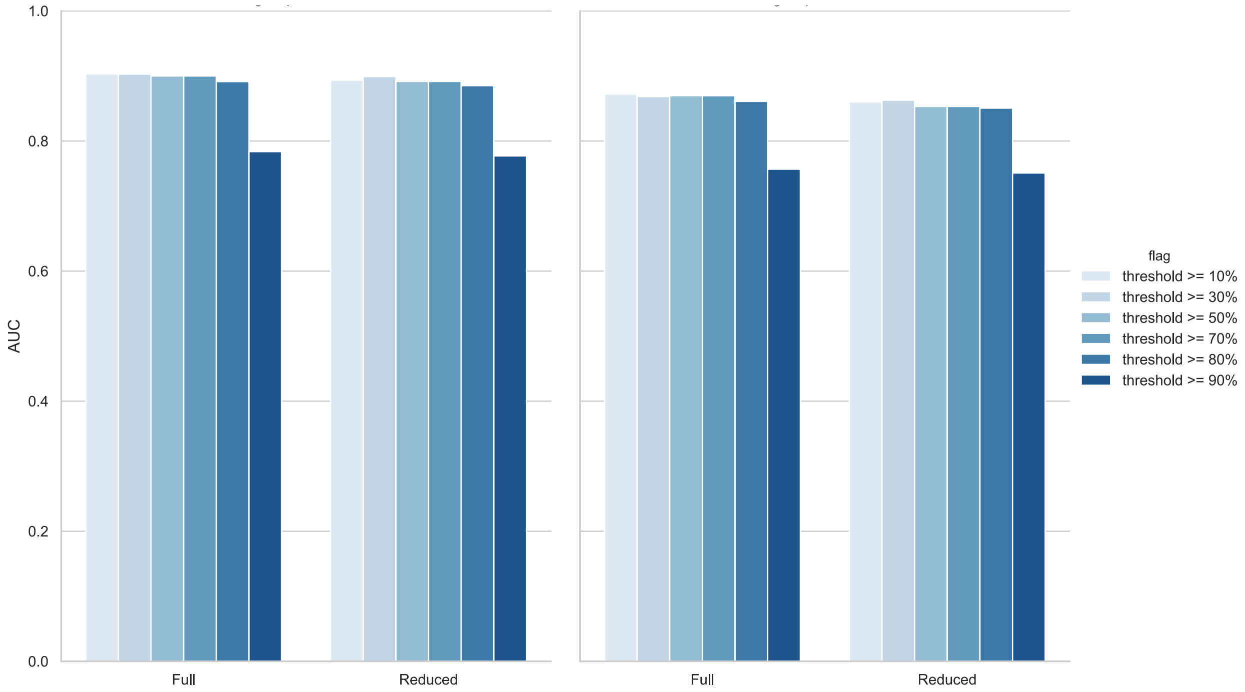

(a)

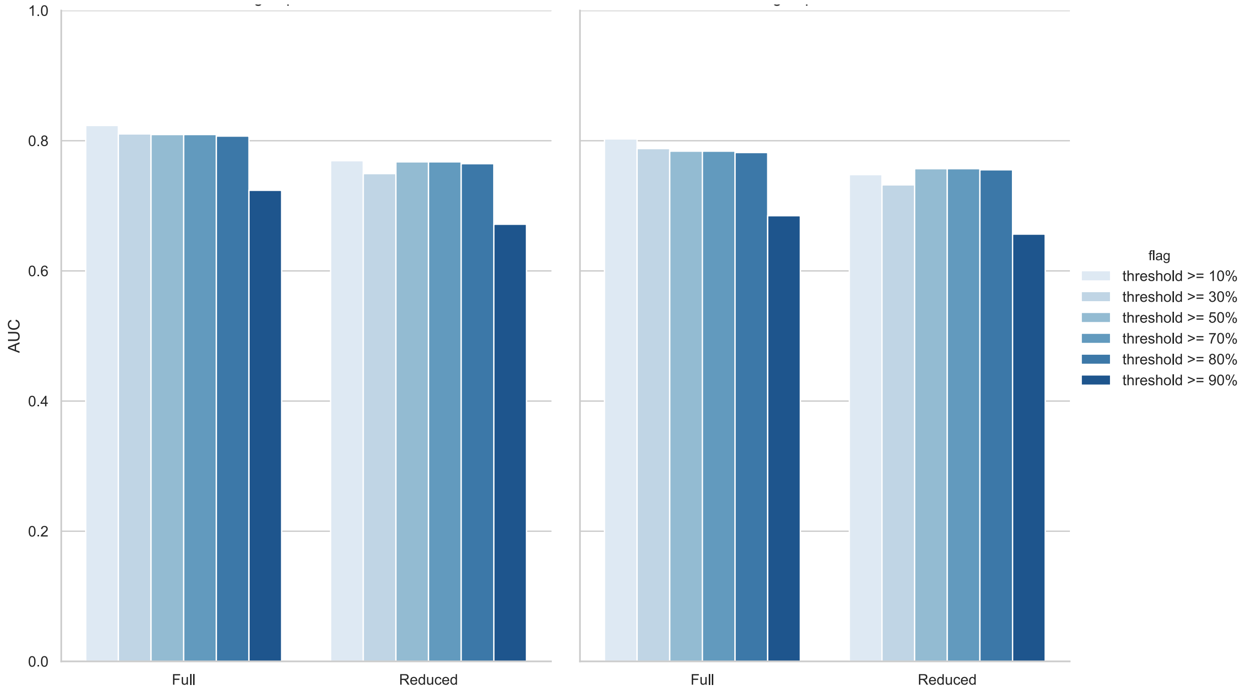

(b)

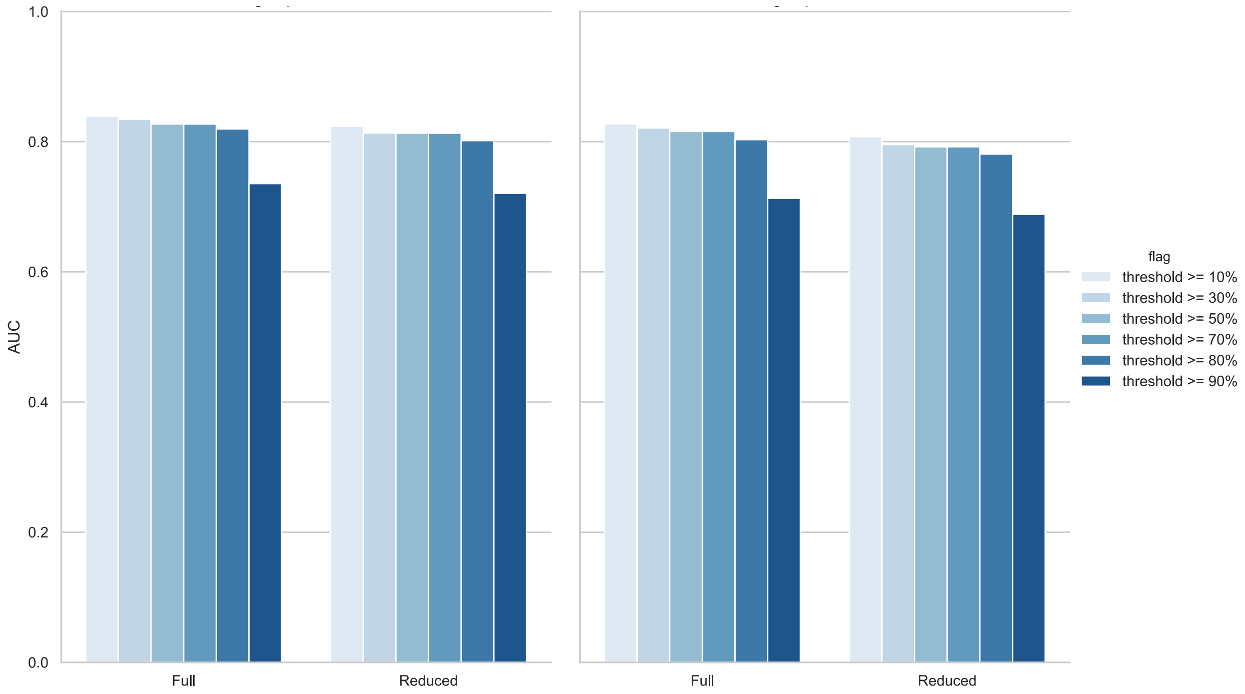
1.0
0.8
0.6
0.4
0.2
0.0
AUC
Full
Reduced
Full
Reduced
flag
threshold >= 10%
threshold >= 30%
threshold >= 50%
threshold >= 70%
threshold >= 80%
threshold >= 90%

(c)

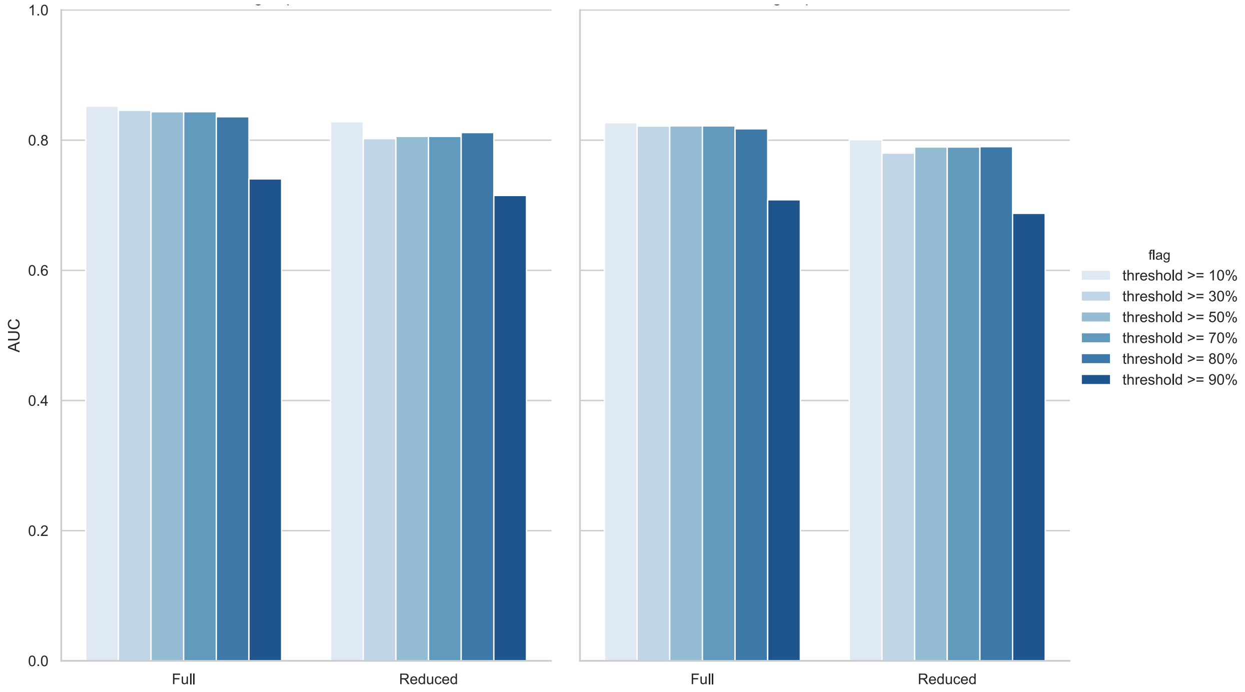
1.0
0.8
0.6
0.4
0.2
0.0
AUC
Full
Reduced
Full
Reduced
flag
threshold >= 10%
threshold >= 30%
threshold >= 50%
threshold >= 70%
threshold >= 80%
threshold >= 90%

(d)
