# Supplementary material for: The Development and Validation of Simplified Machine Learning Algorithms to Predict Prognosis of Hospitalized Patients With COVID-19: Multicenter, Retrospective Study
Source: J Med Internet Res. 2022 Jan 21;24(1):e31549. doi: 10.2196/31549 (PMC8785956; doi:10.2196/31549)

Multimedia Appendix 11. SHAP dependence plot between age and four outcomes (top left: 28-day mortality; top right: composite of 28-day ARDS and respiratory failure; bottom left: 28-day ICU admission; bottom right: 28-day ECMO or ventilator. The features are colored by (a) minimum SpO_2 on admission; (b) respiration rate; (c) lymphocyte count; (d) BUN.

(a) Minimum SpO_2 on admission

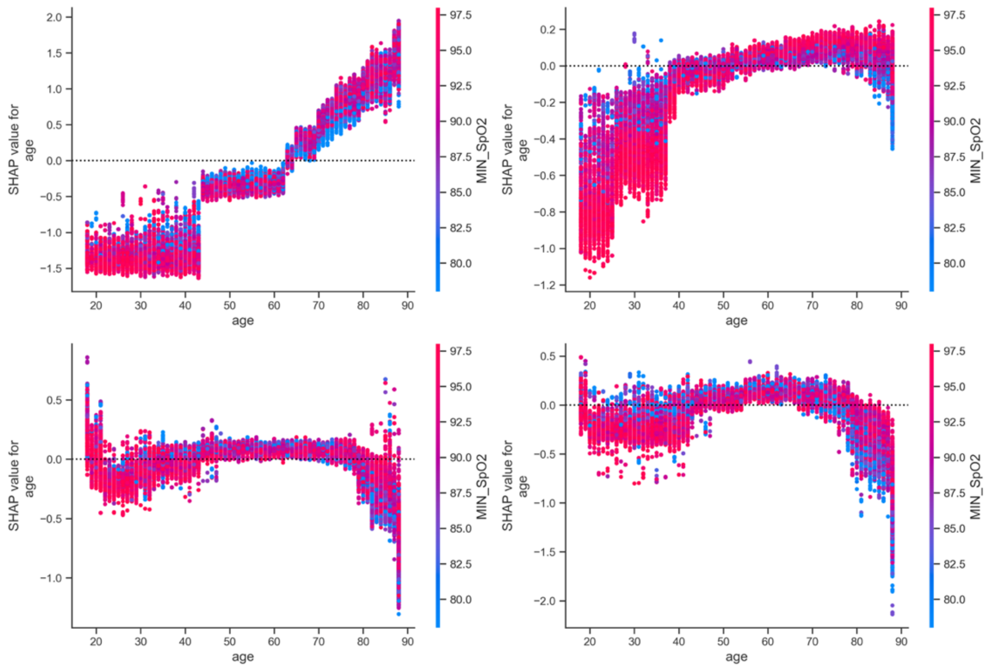

(b) Respiration Rate

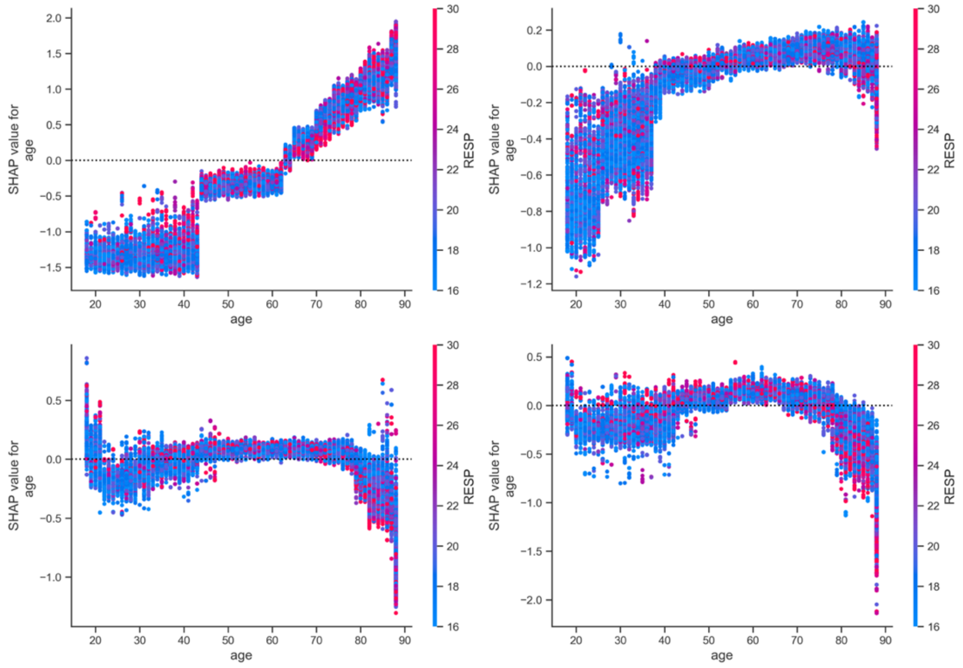

(c) Lymphocyte Count

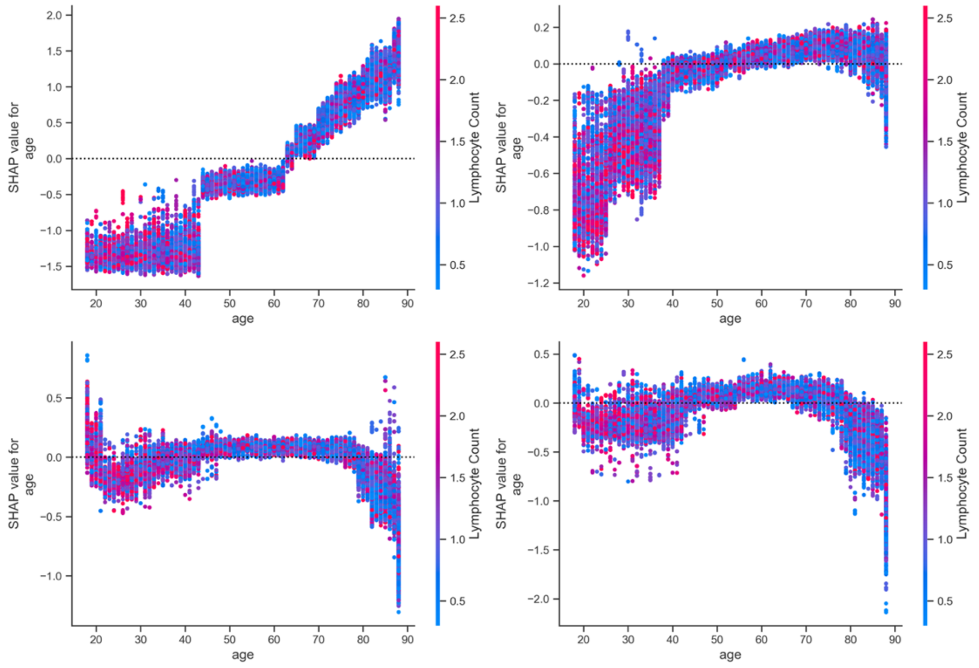

(d) BUN

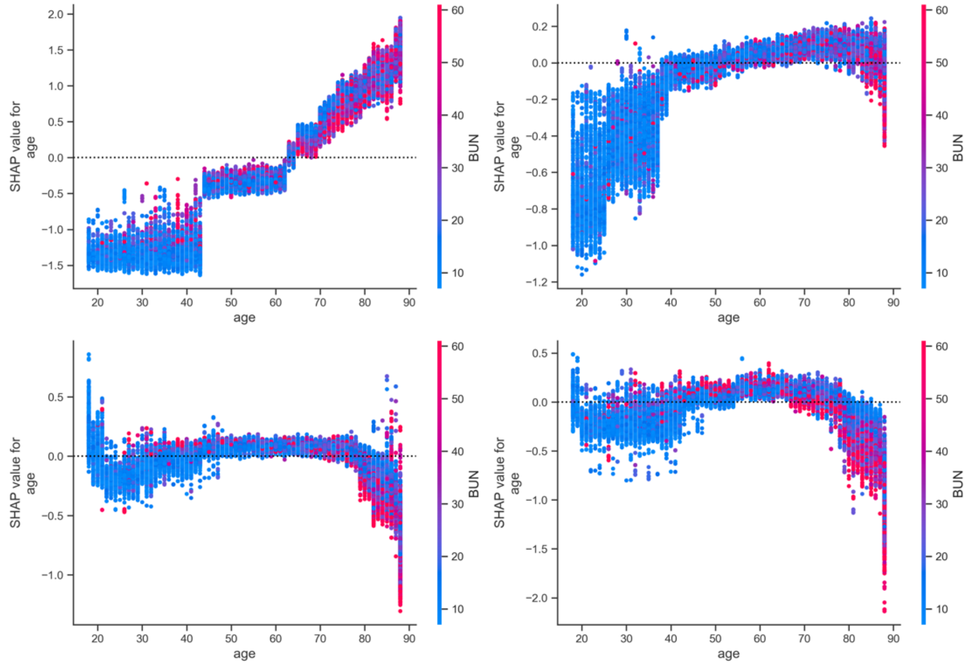

Supplement: Multimedia Appendix 11 [file jmir_v24i1e31549_app11.pdf]
