# Supplementary material for: The Development and Validation of Simplified Machine Learning Algorithms to Predict Prognosis of Hospitalized Patients With COVID-19: Multicenter, Retrospective Study
Source: J Med Internet Res. 2022 Jan 21;24(1):e31549. doi: 10.2196/31549 (PMC8785956; doi:10.2196/31549)
Supplement: Multimedia Appendix 12 [file jmir_v24i1e31549_app12.pdf]

Multimedia Appendix 12. GitLab Repository.

The respective codes and models are hosted at https://gitlab-nimbusglobal.devops.amgen.com/fhe01/covid19_prognosis_jmir
